# RNA binding protein HuR protects against NAFLD by suppressing long noncoding RNA H19 expression

**DOI:** 10.1186/s13578-022-00910-7

**Published:** 2022-10-12

**Authors:** Yanyan Wang, Yun-Ling Tai, Grayson Way, Jing Zeng, Derrick Zhao, Lianyong Su, Xixian Jiang, Kaitlyn G. Jackson, Xuan Wang, Emily C. Gurley, Jinze Liu, Jinpeng Liu, Weidong Chen, Xiang-Yang Wang, Arun J. Sanyal, Phillip B. Hylemon, Huiping Zhou

**Affiliations:** 1 Department of Microbiology & Immunology, Virginia Commonwealth University School of Medicine, 1220 East Broad Street, MMRB-5044, Richmond, VA 23298-0678 USA; 2 McGuire Veterans Affairs Medical Center, Virginia Commonwealth University, Richmond, VA USA; 3 School of Pharmaceutical Science, Anhui University of Chinese Medicine, Hefei, China; 4 Center for Clinical and Translational Research, Virginia Commonwealth University, Richmond, VA 23298 USA; 5 Department of Biostatistics, Virginia Commonwealth University, Richmond, VA USA; 6 Department of Computer Science, University of Kentucky, Lexington, KY USA; 7 Department of Human & Molecular Genetics, Virginia Commonwealth University School of Medicine, Richmond, VA USA; 8 Institute of Molecular Medicine, Virginia Commonwealth University School of Medicine, Richmond, VA USA; 9 Massey Cancer Center, Virginia Commonwealth University School of Medicine, Richmond, VA USA; 10 Department of Internal Medicine/GI Division, Virginia Commonwealth University School of Medicine, Richmond, VA USA

**Keywords:** Bile acids, NASH, Inflammation, Sphingosine kinase, Sphingosine-1 phosphate receptor 2

## Abstract

**Background:**

NAFLD has become the most common chronic liver disease worldwide. Human antigen R (HuR), an RNA-binding protein, is an important post-transcriptional regulator. HuR has been reported as a key player in regulating lipid homeostasis in the liver and adipose tissues by using tissue-specific HuR knockout mice. However, the underlying mechanism by which hepatocyte-specific HuR regulates hepatic lipid metabolism under metabolic stress remains unclear and is the focus of this study.

**Methods:**

Hepatocyte-specific HuR deficient mice (HuR^hKO^) and age-/gender-matched control mice, as well as long-noncoding RNA H19 knockout mice (H19^−/−^), were fed a Western Diet plus sugar water (WDSW). Hepatic lipid accumulation, inflammation and fibrosis were examined by histology, RNA transcriptome analysis, qRT–PCR, and Western blot analysis. Bile acid composition was measured using LC–MS/MS.

**Results:**

Hepatocyte-specific deletion of HuR not only significantly increased hepatic lipid accumulation by modulating fatty acid synthesis and metabolism but also markedly induced inflammation by increasing immune cell infiltration and neutrophil activation under metabolic stress. In addition, hepatic deficiency of HuR disrupted bile acid homeostasis and enhanced liver fibrosis. Mechanistically, HuR is a repressor of H19 expression. Analysis of a recently published dataset (GSE143358) identified H19 as the top-upregulated gene in liver-specific HuR knockout mice. Similarly, hepatocyte-specific deficiency of HuR dramatically induced the expression of H19 and sphingosine-1 phosphate receptor 2 (S1PR2), but reduced the expression of sphingosine kinase 2 (SphK2). WDSW-induced hepatic lipid accumulation was alleviated in H19^−/−^ mice. Furthermore, the downregulation of H19 alleviated WDSW-induced NAFLD in HuR^hKO^ mice.

**Conclusions:**

HuR not only functions as an RNA binding protein to modulate post-transcriptional gene expression but also regulates H19 promoter activity. Hepatic HuR is an important regulator of hepatic lipid metabolism via modulating H19 expression.

**Supplementary Information:**

The online version contains supplementary material available at 10.1186/s13578-022-00910-7.

## Background

Nonalcoholic fatty liver disease (NAFLD) is one of the most common liver diseases that affect more than 25% of the adult population globally, imposing a substantial social and economic burden [1]. NAFLD refers to a broad spectrum of histological conditions, from simple hepatic steatosis (NAFL) to nonalcoholic steatohepatitis (NASH). Moreover, about 10–25% of NAFLD patients will develop NASH, which is now a leading cause of progression to advanced fibrosis, cirrhosis, and hepatocellular carcinoma (HCC) [2]. Furthermore, NASH–HCC is the second leading cause of liver transplantation related to HCC in the United States [3, 4]. Over the last several decades, considerable efforts have been made to identify the potential molecular mechanisms underlying NAFLD progression. In addition to demographic and genetic factors, obesity, diabetes, and cardiovascular diseases are also closely associated with NASH–HCC [5, 6]. However, the pathogenesis of NAFLD remains incompletely understood, and no effective treatment is available.

It has been well accepted that the progression of NAFLD is driven by multiple factors, such as dysregulation of hepatic lipid and bile acid metabolism, activation of inflammatory pathways and endoplasmic reticulum (ER) stress response, as well as dysbiosis of the gut microbiome [2, 7]. We and others have previously reported that sphingosine-1 phosphate receptor 2 (S1PR2) and sphingosine kinase 2 (SphK2) are important regulators of hepatic lipid metabolism [8–10]. It also has been reported that long noncoding RNA (lncRNA) H19, an imprinted and maternally expressed gene, is an important regulator in cholestatic liver fibrosis, inflammation, and hepatic lipid metabolism [11–14]. The aberrant expression of H19 is associated with liver fibrosis in HCC patients [15].

Human antigen R (HuR), also known as HuA and embryonic lethal abnormal vision-like 1 (ELAVL1), functions to regulate the expression of coding and noncoding RNAs by post-transcriptional mechanisms [16, 17]. Global HuR-knockout mice exhibited embryonic lethality due to extra-embryonic placental defects [18]. Recently, the importance of HuR-mediated roles in cell signaling, inflammation, fibrogenesis, and cancer development in the liver has attracted a great deal of attention [19]. Several studies have reported that hepatic HuR protects against NAFLD by targeting lipid and glucose metabolism, regulating lipid transport, and inhibiting adipogenesis [20–22]. A recent study reported that HuR is a gatekeeper of liver homeostasis and liver-specific deletion of HuR accelerated NASH fibrosis and HCC [20]. However, the hepatocyte-specific roles of HuR in NAFLD pathogenesis have not been fully explored, and the underlying mechanisms remain largely unclear.

In the present study, hepatocyte-specific HuR knockout (HuR^hKO^) mice were generated to evaluate the role of HuR in regulating hepatic lipid metabolism using a Western diet plus sugar water (WDSW)-induced NAFLD mouse model. The current study shows that HuR^hKO^ mice exacerbate the progression of WDSW-induced NAFLD as indicated by enhanced liver steatosis, inflammation, and fibrosis. Mechanistically, hepatocyte-specific HuR deficiency upregulated the expression of H19, which promoted inflammation and hepatic lipid accumulation. In addition, hepatocyte-specific HuR deficiency disrupted bile acids homeostasis. These results suggested that hepatocyte HuR may be a potential therapeutic target for NAFLD.

## Results

### Hepatocyte-specific HuR deficiency enhances WDSW-induced NAFLD

**Fig. 1:**
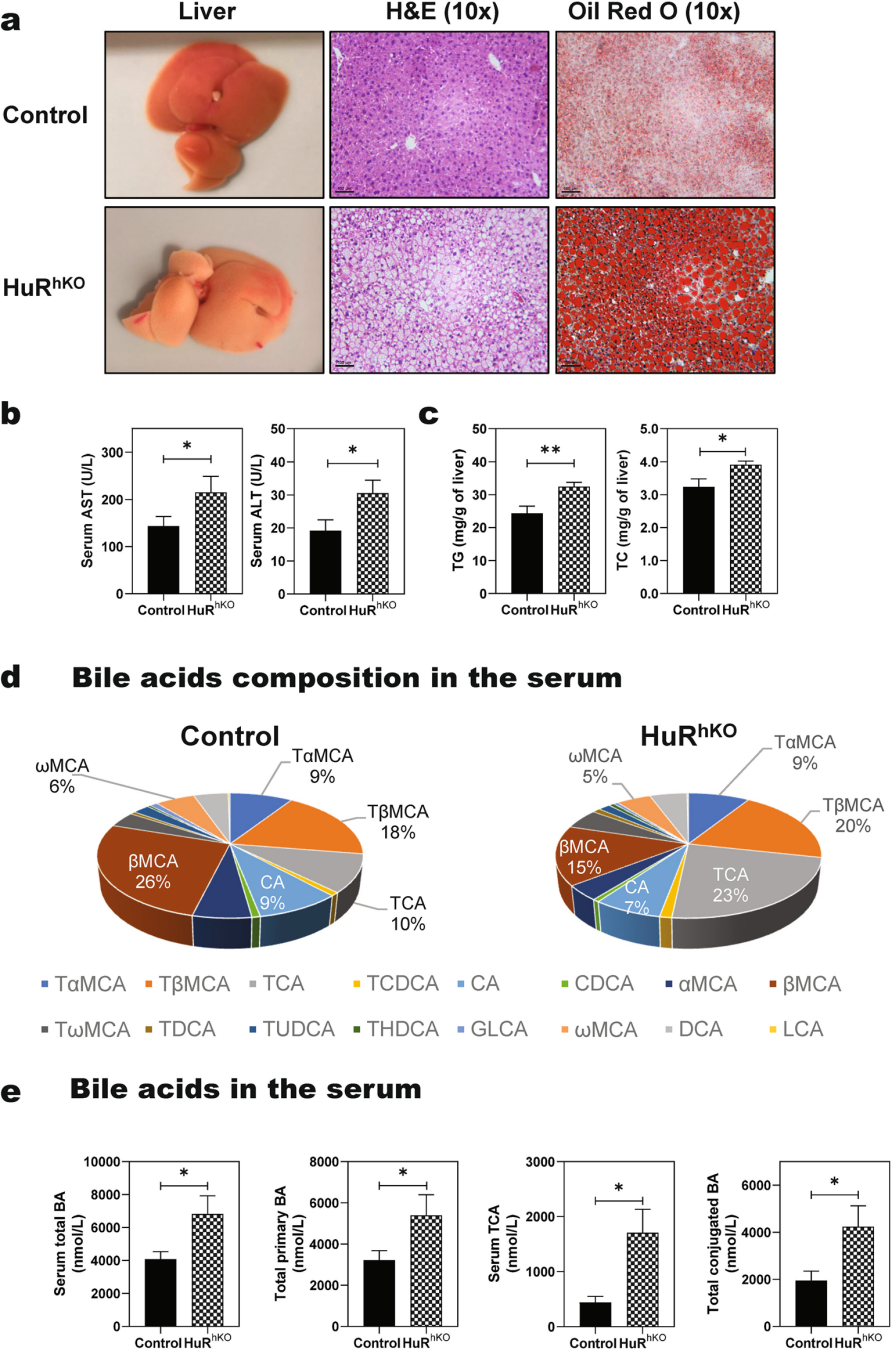
Hepatocyte-specific HuR deficiency enhances WDSW-induced NAFLD. Age and gender-matched HuR^hKO^ and control mice were fed ad libitum a high-fat diet with 42% kcal from fat and containing 0.1% cholesterol plus a high fructose-glucose solution (23.1 g/L d-fructose + 18.9 g/L d-glucose) (Western diet plus sugar water, WDSW) for 4 weeks. **a** Representative image of the liver and the liver sections stained with H&E and Oil red O staining (scale bar, 100 μm for 10 × magnification). **b** Serum liver enzyme levels (AST, ALT). **c** Hepatic triglyceride (TG) and total cholesterol (TC). **d** Bile acid composition profile in the serum is expressed by % of total bile acids. **e** Total bile acids (BA), total primary BA, TCA, and total conjugated BA in the serum. Data are expressed as mean ± SEM. Statistical significance: *p < 0.05 vs Control, n = 10–12 (both male and female)

Although two recent studies have reported the hepatic-specific role of HuR modulating lipid metabolism in mouse NAFLD models, both studies used liver-specific HuR knockout mice by cross-breeding a *HuR*^*flox/flox*^ mouse with albumin-Cre mice, not hepatocyte-specific knockout mice [20, 22]. To delineate the hepatocyte-specific role of HuR in the NAFLD disease progression, HuR^hKO^ mice were generated by tail-vein injection of HuR^flox/flox^ mice with AAV8-TBGP-Cre recombinase and AAV8-TBGP-GFP was used as a control. HuR^hKO^ and control mice were fed ad libitum a WDSW for 4 weeks. As shown in Additional file 1: Fig.S1a–c, the HuR mRNA and protein levels were significantly reduced in the liver of HuR^hKO^ mice. Immunofluorescence staining further confirmed the deletion of HuR in hepatocytes (Additional file 1: Fig. S1d). As shown in Fig. 1a, b, hepatocyte-specific deletion of HuR exacerbated WDSW-induced hepatic lipid accumulation and liver injury following 4-week feeding as indicated by increased lipid accumulation (H&E and Oil Red O staining), and increased serum aspartate aminotransferase (AST) and alanine aminotransferase (ALT) levels. Although the liver index (ratio of liver/body weight), serum cholesterol, triglycerides, glucose, and alkaline phosphatase (ALP) levels were not significantly altered (Additional file 1: Fig. S1e–g), hepatic levels of triglycerides and cholesterol in HuR^hKO^ mice were much higher than those in control mice (Fig. 1c). However, no significant change was noticed in the serum ALT and AST in HuR^hKO^ and control mice under a normal diet (Additional file 1: Fig. S1h, i), which was consistent with the H&E staining (Additional file 1: Fig. S1j).

Previous studies have reported that NALFD disease severity is associated with specific changes in circulating bile acids in human NASH patients and mouse NASH models. Serum taurocholic acid (TCA) level is significantly increased in NASH patients [23, 24]. To examine whether the serum bile acid profile was changed in HuR^hKO^ mice after 4-week WDSW feeding, serum bile acid composition and levels were measured using LC–MS/MS [24]. As shown in Fig. 1d, the percentage of TCA in total bile acids was significantly increased from 10% (Control) to 23% (HuR^hKO^), while βMCA was significantly decreased from 26% (Control) to 15% (HuR^hKO^) after 4-week WDSW feeding. Total serum bile acid levels were significantly increased in HuR^hKO^ mice; including, total primary, secondary, and conjugated bile acids (Fig. 1e and Additional file 1: Table S2). In addition, serum levels of TCA, TβMCA, TCDCA, TωMCA, and THDCA in HuR^hKO^ mice were much higher than those in control mice (Additional file 1: Table S3).

### Hepatocyte-specific HuR deficiency enhances WDSW-induced NAFLD by modulating the global transcriptomic profile

**Fig. 2:**
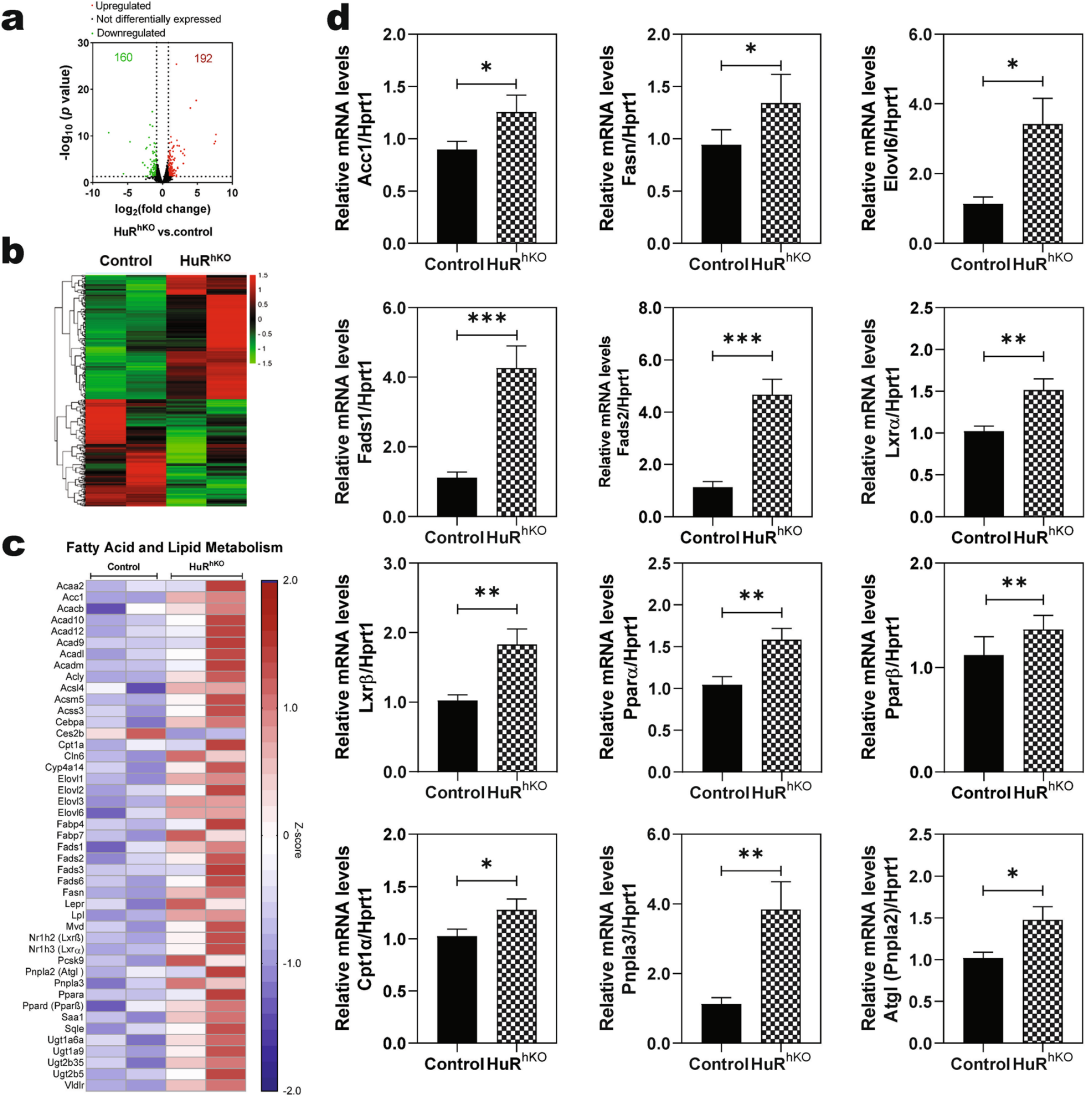
Hepatocyte-specific HuR deficiency enhances WDSW-induced NAFLD by modulating the global transcriptomic profile. Total liver RNA of HuR^hKO^ and control mice fed with WDSW for 4 weeks was processed for transcriptome sequencing (RNA-Seq). Significantly up-or down-regulated genes were determined as fold change ≥ 1.8 and p-value < 0.05. **a** Volcano plots in HuR^hKO^ *vs.* control group. Red dots indicate upregulated genes; green dots indicate downregulated genes; black dots indicate not differentially expressed genes. **b** Hierarchical clustering heatmaps for differentially expressed genes in HuR^hKO^ *vs.* control group. A Z-score was calculated for the RNA-Seq data to normalize tag counts. Red and green colors indicate up- and down-regulated gene expression, respectively. **c** Representative heatmap of the key genes involved in fatty acid and lipid metabolism in the liver of HuR^hKO^ *vs.* control group. **d** Relative mRNA levels of the key genes involved in fatty acid and lipid metabolism: Acc1, Fasn, Elovl6, Fads1, Fads2, Lxrα, Lxrβ, Pparα, Pparβ, Cpt1α, Pnpla3, and Pnpla2 (Atgl). The mRNA levels were determined by real-time RT–PCR and normalized with HPRT1 as an internal control. Data are expressed as the mean ± SEM. Statistical significance relative to control: * p < 0.05, ** p < 0.01, *** p < 0.001 (n = 10–12, male and female)

To illustrate the underlying mechanisms by which hepatic HuR deficiency-induced NAFLD disease progression, we performed RNA-seq transcriptome analysis. As shown in Fig. 2a, b, WDSW-feeding induced the upregulation of 192 genes and down-regulation of 160 genes in HuR^hKO^ mice compared to the control mice. Gene Ontology analysis showed that WDSW-feeding significantly impacted the major pathways in biological process (BP), cellular component (CC), and molecular function (MF) related to metabolic processes and immunological responses (Additional file 1: Fig. S2a–c) in HuR^hKO^ mice compared to the control mice, including cholesterol metabolic process, biosynthetic process, inflammatory response, extracellular exosome, lipoprotein, extracellular matrix, oxidoreductase activity, fatty acid-binding, etc. Furthermore, KEGG pathways analysis showed that metabolic pathways, including steroid hormone biosynthesis and inflammatory response, were dysregulated in HuR^hKO^ mice (Additional file 1: Fig. S2d).

### Hepatocyte-specific HuR deficiency enhances WDSW-induced dysregulation of fatty acid and lipid metabolism

Dysregulation of lipid metabolism contributes to the development of NAFLD [25]. As shown in Fig. 2c, most of the genes involved in the fatty acid biosynthesis pathway were increased in HuR^hKO^ mice compared to the control mice after 4 weeks of WDSW feeding; including acetyl CoA carboxylase (Acc1), fatty acid synthase (Fasn), elongation of very-long-chain fatty acids member 6 (Elovl6), fatty acid desaturases (Fads1&2), liver X receptor (Lxrα&β), peroxisome proliferator-activated receptor (Pparα&β), carnitine palmitoyltransferase 1 (Cpt1α), patatin-like phospholipase domain containing (Pnpla3), and adipose triglyceride lipase (Atgl, also known as Pnpla2), etc. The mRNA expression levels of key genes involved in hepatic lipid metabolism were further confirmed by real-time RT–PCR. As shown in Fig. 2d, the mRNA levels of Acc1, Fasn, Elovl6, Fads1, Fads2, Lxrα, Lxrβ, Pparα, Pparβ, Cpt1α, Pnpla3, and Atgl were significantly increased in HuR^hKO^ mice compared to the control mice after 4-weeks of WDSW feeding.

### Hepatocyte-specific HuR deficiency enhances WDSW-induced inflammation and oxidative stress

**Fig. 3:**
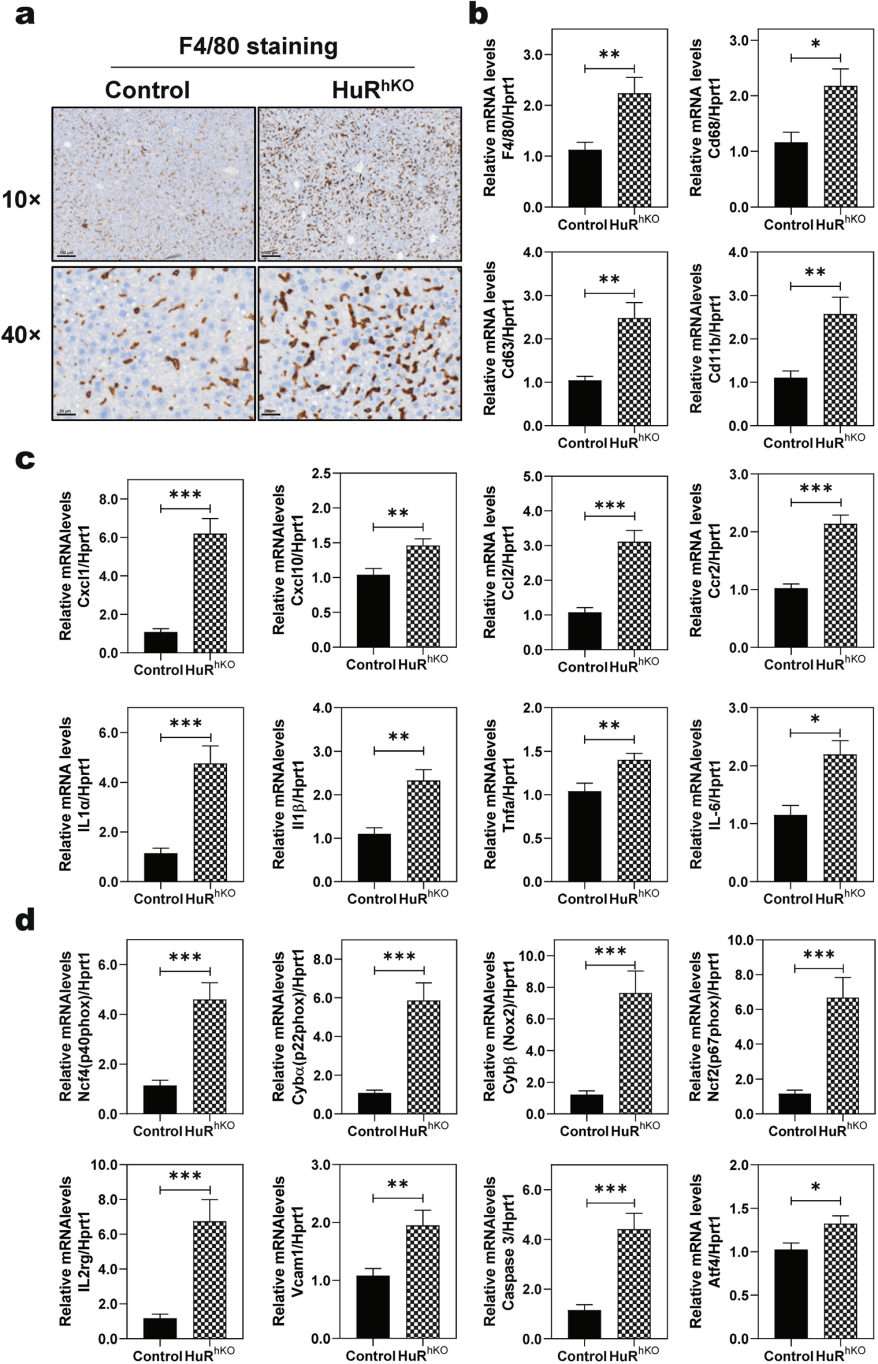
Hepatocyte-specific HuR deficiency enhances WDSW-induced inflammation and oxidative stress. **a** Representative images of immunohistochemistry (IHC) staining of F4/80 (scale bar, 100 μm for 10 × and 20 μm for 40 × magnification). Relative mRNA levels of genes involved in inflammation and oxidative stress were determined by real-time RT–PCR and normalized with HPRT1 as an internal control. **b** Macrophage markers: F4/80, Cd68, Cd63, and Cd11b; **c** Chemokines: Cxcl1, Cxcl10, Ccl2, and Ccr2; Inflammatory cytokines: IL1α, IL1β, Tnfα, and IL-6; **d** Neutrophil activation: Ncf4, Cybα, Cybβ, Ncf2, IL2rg, and Vcam1; Stress: Caspase 3 and Atf4. Data are expressed as the mean ± SEM. Statistical significance relative to control: * p < 0.05, ** p < 0.01, *** p < 0.001 (n = 12, male and female)

Inflammation and stress response are important driving forces in promoting NAFL to NASH progression [26–28]. RNA-seq analysis showed that the key genes involved in inflammatory and stress responses, such as F4/80, Cd68, Cd63, C-X-C Motif Chemokine Ligand 1 (Cxcl1), Cxcl10, chemokine ligand 2 (Ccl2), C–C chemokine receptor type 2 (Ccr2), Caspase 3, Activating transcription factor 4 (Atf4), interleukin 1α (IL-1α), were significantly increased in HuR^hKO^ mice fed with a WDSW for 4 weeks (Additional file 1: Fig. S3a). Hepatocyte-specific deletion of HuR promoted WDSW-induced macrophage infiltration to the liver as indicated by the IHC staining of F4/80 antigen; a mature cell surface glycoprotein expressed at high levels on various macrophages (Fig. 3a). Real-time PCR results further showed that the mRNA expression levels of major marker genes of macrophages, inflammatory cytokines and chemokines, such as F4/80, Cd68, Cd63, Integrin alpha M (also known as Cd11b) (Fig. 3b), Cxcl1, Cxcl10, Ccl2, Ccr2, IL-1α, IL-1β, tumor necrosis factor α (Tnfα), and IL-6, were significantly increased (Fig. 3c).

Neutrophils, the most abundant leukocytes in circulation, play a vital role in innate immunity [29]. However, inappropriate activation of neutrophils can cause tissue damage, which has been involved in different diseases, including various liver diseases [30, 31]. Our recent study reported that in the WDSW-induced NASH mouse model, the expression levels of major genes involved in neutrophil activation were significantly upregulated in the liver [24]. As shown in Additional file 1: Fig. S3b, the key genes involved in neutrophil activation, such as NADPH oxidase 2 [Nox2, also known as neutrophil cytochrome b heavy chain (Cybβ), or p91phox], neutrophil cytosolic factor 2 (Ncf2, also known as p67phox), Ncf4 (also known as p40phox), Cybα (also known as p22phox), IL-2 receptor gamma unit (IL2rg), intercellular adhesion molecule 1 (ICAM1) and vascular cell adhesion molecule 1 (vcam1), were significantly upregulated in the liver of WDSW-fed HuR^hKO^ mice. The upregulation of mRNA levels was further confirmed by real-time RT–PCR (Fig. 3d). Pathway analysis further showed that oxidative phosphorylation in mitochondria and MAPK signaling pathways were significantly dysregulated in WDSW-fed HuR^hKO^ mice compared to control mice (Additional file 1: Fig.S4 and S5).

### HuR is a suppressor of lncRNA H19 transcription

**Fig. 4:**
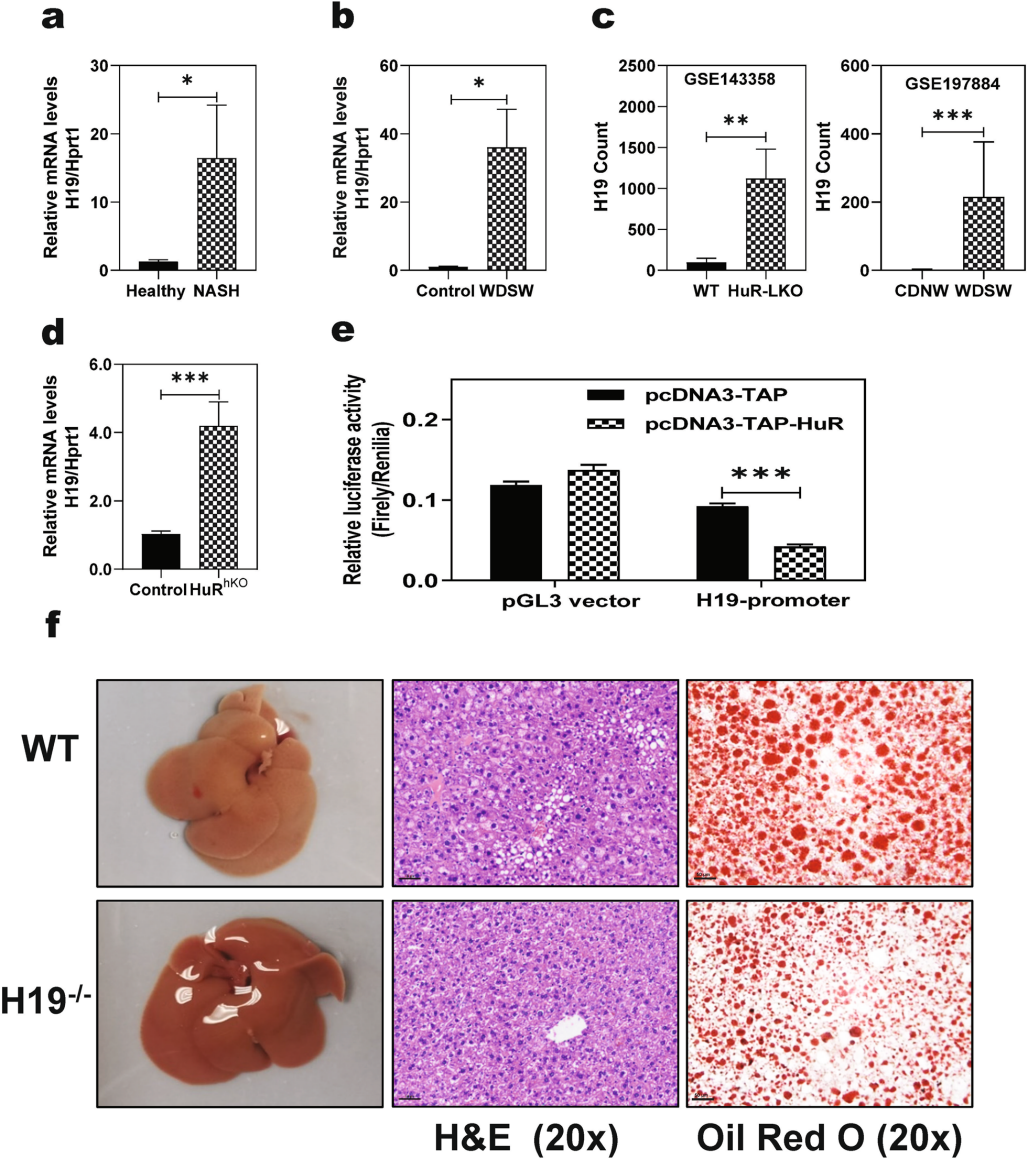
HuR is a suppressor of lncRNA H19 transcription. **a** Relative mRNA levels of lncRNA H19 in the liver of NASH patients and healthy controls were determined by real-time RT–PCR. Statistical significance relative to healthy control, *p < 0.05 (n = 8–10). **b** Relative mRNA levels of H19 in the liver of mice fed a WDSW or control diet for 21 weeks. Statistical significance relative to control mice, *p < 0.05 (n = 5). **c** H19 count from the publically available RNAseq data set (GSE143358) in liver-specific HuR knockout mice; H19 count from the most recent study (GSE197884) in a newly developed preclinical NASH–HCC model with C57/BL6NJ mice. Statistical significance relative to control mice, **p < 0.01 and ***p < 0.001 (n = 3). **d** Relative mRNA levels of H19 in the liver of HuR^hKO^ and control mice fed with WDSW for 4 weeks. Statistical significance relative to control mice, ***p < 0.001 (n = 10–12). **e** Effect of HuR on H19 promoter activity. HEK-293 cells were transfected with pGL3-human H19-promoter or pGL3-control vector together with pcDNA3-TAP-human HuR or pcDNA3-TAP vector along with Renilla control vector. After transfection for 48 h, the luciferase activities were measured using a dual-luciferase reporter assay kit. The promoter activity was expressed by relative luciferase activity using the ratio of firefly luciferase activity to Renilla luciferase activity. Statistical significance relative to control, ***p < 0.001 (n = 5). **f** Representative image of the liver and the liver sections stained with H&E and Oil red O staining (scale bar, 50 µm for 20 × magnification) in H19^−/−^ and WT mice fed with WDSW for 4 weeks

We and others have previously reported that aberrant expression of lncRNA H19 is closely associated with hepatic inflammation and liver fibrosis in various liver diseases, including NASH [12–14]. To further identify the potential underlying mechanisms by which hepatic deletion of HuR promotes NAFLD progression, we examined H19 expression in the livers of human NASH patients and WDSW-induced NASH mouse models. As shown in Fig. 4a, hepatic H19 mRNA levels were increased more than tenfold in human NASH patients compared to healthy controls. Similarly, in NASH mice fed WDSW for 21 weeks, hepatic H19 mRNA levels were increased more than 30-fold compared to control mice (Fig. 4b). Analysis of the publically available RNAseq data set from a most recent study (GSE143358) showed that H19 is the most significantly upregulated gene in liver-specific HuR knockout mice (Fig. 4c) [20]. In a newly developed preclinical NASH–HCC model with C57/BL6NJ mice, H19 is in the top ten upregulated genes (GSE197884) (Fig. 4c) [32]. We also found that H19 was significantly upregulated in the liver of HuR^hKO^ mice fed with WDSW for 4 weeks (Fig. 4d).

To identify the mechanism underlying HuR deficiency-induced H19 expression, a luciferase reporter assay with a human H19 promoter was performed to examine the impact of HuR on H19 transcriptional activity. As shown in Fig. 4e, overexpression of HuR significantly inhibited H19 promoter activity. To further determine the role of H19 in hepatic steatosis and inflammation in vivo, H19^−/−^ mice and gender and age-matched WT mice were fed a WDSW for 4 weeks. As shown in Fig. 4f, H&E and Oil red O staining indicated that H19^−/−^ mice were protected from WDSW-induced hepatic lipid accumulation.

### HuR regulates the expression of SphK2 and S1PR2

**Fig. 5:**
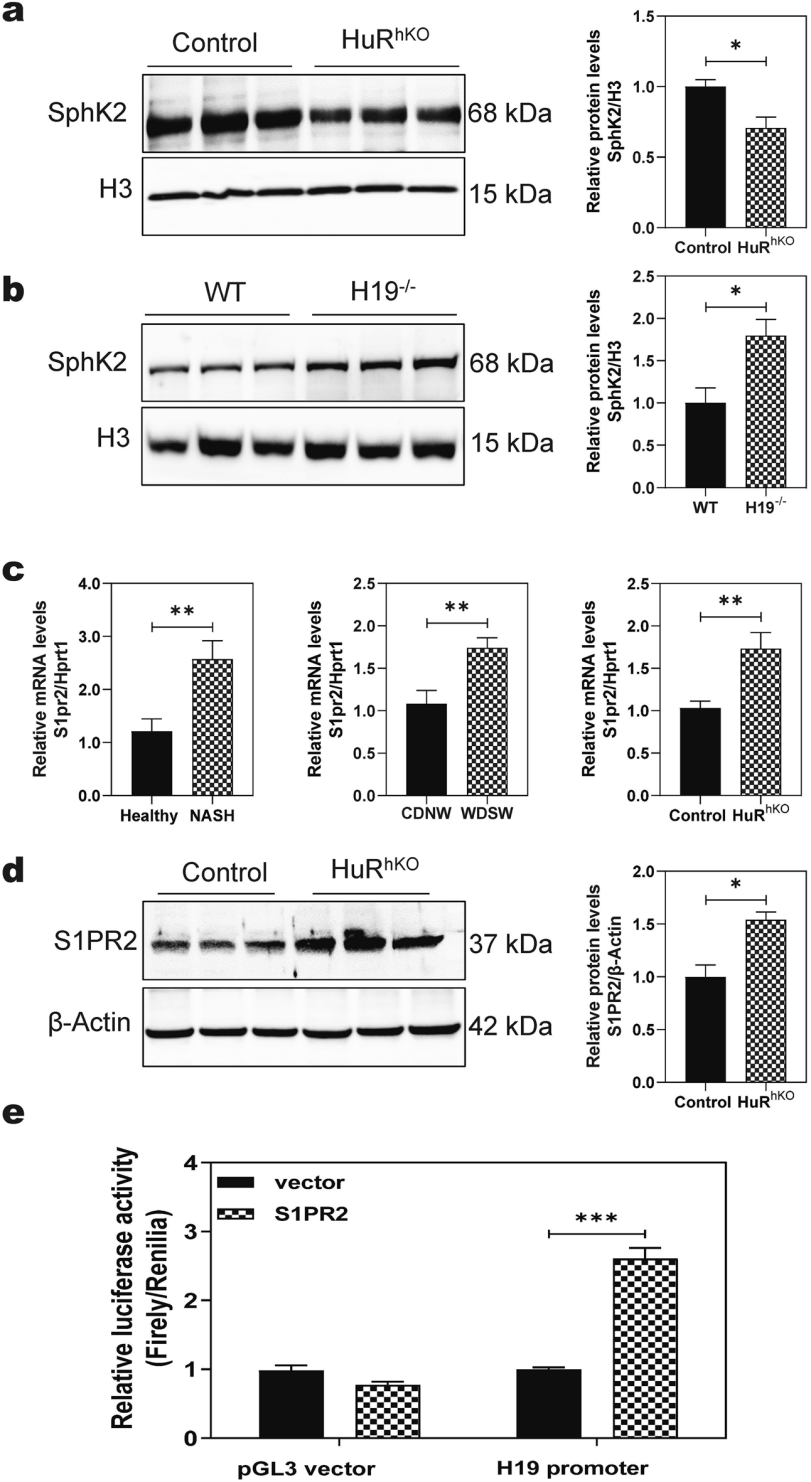
HuR regulates the expression of SphK2 and S1PR2. **a** Representative immunoblot images of nuclear SphK2 in the livers of HuR^hKO^ and control mice fed with WDSW for 4 weeks are shown. The relative protein level of SphK2 was calculated using histone H3 as a loading control. Statistical significance relative to control, *p < 0.05 (n = 3) **b** Representative immunoblot images of nuclear SphK2 in the livers of H19^−/−^ and control mice fed with WDSW for 4 weeks are shown. The relative protein level of SphK2 was calculated using histone H3 as a loading control. Statistical significance relative to control, *p < 0.05 (n = 3). **c** Relative mRNA levels of S1PR2 in the liver of human NASH patients, WDSW-NASH mice, and HuR^hKO^ mice fed with WDSW for 4 weeks, respectively. Statistical significance relative to control, **p < 0.01. **d** Representative immunoblot images of S1PR2 in the liver are shown and normalized with β-actin as an internal control of HuR^hKO^ mice fed with WDSW for 4 weeks. Statistical significance relative to control, *p < 0.05 (n = 3). **e** Effect of S1PR2 on H19 promoter activity. HEK-293 cells were transfected with pGL3-human H19-promoter or pGL3-control vector together with pcDNA3-hS1PR2 or control vector along with Renilla control vector. After transfection for 48 h, the luciferase activities were measured using a dual-luciferase reporter assay kit. The promoter activity was expressed by relative luciferase activity using the ratio of firefly luciferase activity to Renilla luciferase activity. Statistical significance relative to control, ***p < 0.001 (n = 5)

Previous studies have reported that SphK2, a key enzyme in sphingolipid catabolism, plays a critical role in regulating hepatic lipid metabolism [8–10]. Global SphK2 deficient (SphK2^−/−^) mice developed overt fatty liver compared to the control mice on a two-week high-fat diet [10]. SphK2 is primarily located in the cell nucleus. As shown in Fig. 5a, the nuclear protein levels of SphK2 were significantly decreased in the livers of WDSW-fed HuR^hKO^ mice compared to control mice. Deletion of H19 increased SphK2 nuclear protein level (Fig. 5b). However, neither hepatocyte HuR deficiency nor deletion of H19 significantly altered the total protein levels of SphK2 in the livers (Additional file 1: Fig. S6a, b). Our previous studies also reported that S1PR2 deficiency significantly reduced cholestasis-induced cholangiocyte proliferation and liver injury [33]. As shown in Fig. 5c, d, S1PR2 mRNA levels were significantly upregulated in the livers of human NASH patients and the WDSW-induced NASH mouse model. Similarly, both mRNA and protein levels of S1PR2 were significantly increased in WDSW-fed HuR^hKO^ mice. To further elucidate the potential role of S1PR2 in HuR deficiency-induced metabolic liver injury, we examined the effect of overexpression of S1PR2 on H19 promoter activity using the luciferase report assay. As shown in Fig. 5e, S1PR2 significantly enhanced H19 promoter activity.

### Hepatocyte-specific HuR deficiency enhances WDSW-induced dysregulation of bile acid homeostasis

**Fig. 6:**
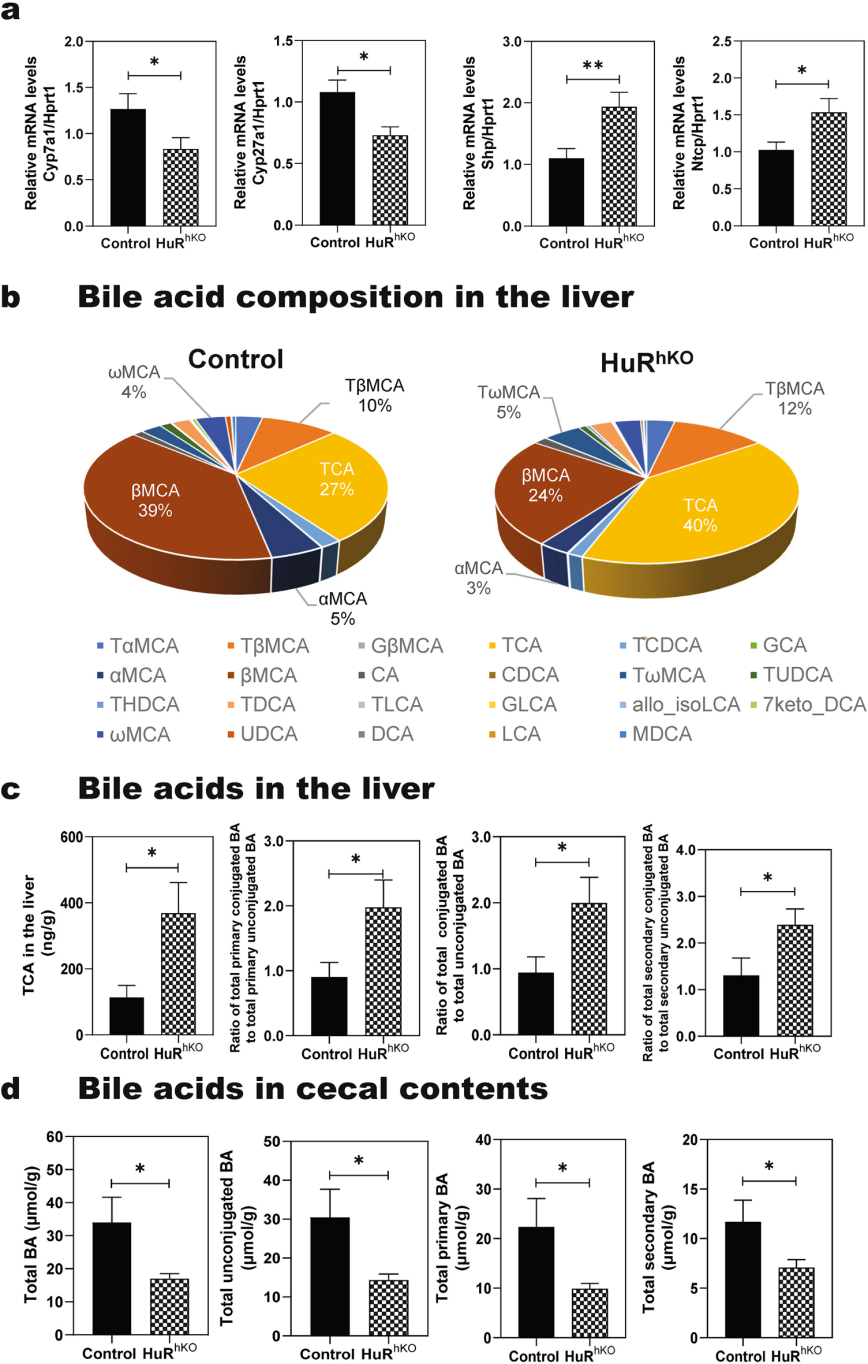
Hepatocyte-specific HuR deficiency enhances WDSW-induced dysregulation of bile acid homeostasis. **a** Relative mRNA levels of Cyp7a1, Cyp27a1, Shp, and Ntcp are shown and normalized with HPRT1 as an internal control in the liver of HuR^hKO^ vs. Control mice fed with WDSW for 4 weeks. **b** BA composition profile in the liver is expressed by % of total BA. **c** TCA, the ratio of total primary conjugated BA to total primary unconjugated BA, the ratio of total conjugated BA to total unconjugated BA, and the ratio of total secondary conjugated BA to total secondary unconjugated BA in the liver. **d** Total BA, total unconjugated BA, total primary BA, and total secondary BA in the cecal contents. Data are expressed as the mean ± SEM. Statistical significance relative to control: * p < 0.05, ** p < 0.01, n = 8–12

Bile acids are critical in regulating hepatic lipid, glucose, and energy metabolism as they are important signaling molecules [24, 34, 35]. LC–MS/MS analysis of serum bile acid levels indicated that hepatocyte-specific HuR deficiency aggravated WDSW-induced disruption of bile acid homeostasis (Fig. 1d, e, Additional file 1: Table S2 and S3). Real-time PCR analysis showed that the expression levels of two rate-limiting enzymes in the bile acid synthesis pathway, cholesterol 7 alpha-hydroxylase (Cyp7α1) and cholesterol 27 alpha-hydroxylase (Cyp27α1), were significantly decreased. In contrast, the expression levels of small heterodimer partner (Shp) and Na + -taurocholate cotransporting polypeptide (Ntcp) were significantly upregulated in the livers of WDSW-fed HuR^hKO^ (Fig. 6a). To further determine the impact of hepatocyte-specific deletion of HuR on enterohepatic circulation, the bile acid composition and levels in the liver, intestinal ileum, and cecal contents were measured using LC–MS/MS. As shown in Fig. 6b, the percentage of TCA in total hepatic bile acids was increased from 27% (Control) to 40% (HuR^hKO^), while βMCA was decreased from 39% (Control) to 24% (HuR^hKO^). Although the total liver bile acids showed no significant changes between the WDSW-fed HuR^hKO^ mice and control mice, the TCA level, the ratio of total primary conjugated bile acids to total primary unconjugated bile acids, the ratio of total conjugated bile acids to total unconjugated bile acids, and the ratio of total secondary conjugated bile acids to total secondary unconjugated bile acids were increased in HuR^hKO^ mice (Fig. 6c, Additional file 1: Table S4 and S5). Moreover, it should be noted that TCA has been shown to be a potent activator of S1PR2 [36]. In the intestinal ileum, as shown in Additional file 1: Fig. S7a, the percentage of TCA in total bile acids was increased from 27% (Control) to 35% (HuR^hKO^). Hepatocyte HuR deficiency had no significant effect on intestinal total bile acids, the ratio of total primary conjugated bile acids to total primary unconjugated bile acids, and the ratio of total conjugated bile acids to total unconjugated bile acids in the intestine (Additional file 1: Fig. S7b and Additional file 1: Table S6, S7). However, hepatocyte HuR deficiency significantly reduced bile acid levels, including total bile acids, total unconjugated bile acids, total primary bile acids, and total secondary bile acids in the cecal contents (Fig. 6d, Additional file 1: Fig. S7c, d, and Additional file 1: Tables S8, S9). Under normal diet feeding conditions, the deletion of HuR in hepatocytes significantly changed bile acid levels and composition in the serum. As shown in Additional file 1: Fig. S8a, the percentages of TCA and TβMCA in total bile acids increased from 14 to 63% and 3% to 20%, respectively, in normal diet fed HuR^hko^ mice. While the percentage of βMCA decreased from 25 to 1%. The total bile acid level and ratios of total primary bile acid to total bile acid and total primary bile acid to total secondary bile acids were also increased in the serum of normal diet-fed HuR^hko^ mice (Additional file 1: Tables S10, S11). There were no significant changes in hepatic bile acid level and composition in normal diet-fed HuR^hko^ mice (Additional file 1: Fig. S8b, Tables S12, S13). However, the total bile acid levels in the intestine and cecal contents were reduced, although the bile acid composition was not significantly changed in normal diet-fed HuR^hko^ mice (Additional file 1: Tables S14–17; Fig. S9).

### Hepatocyte-specific HuR deficiency enhances WDSW-induced hepatic fibrosis

**Fig. 7:**
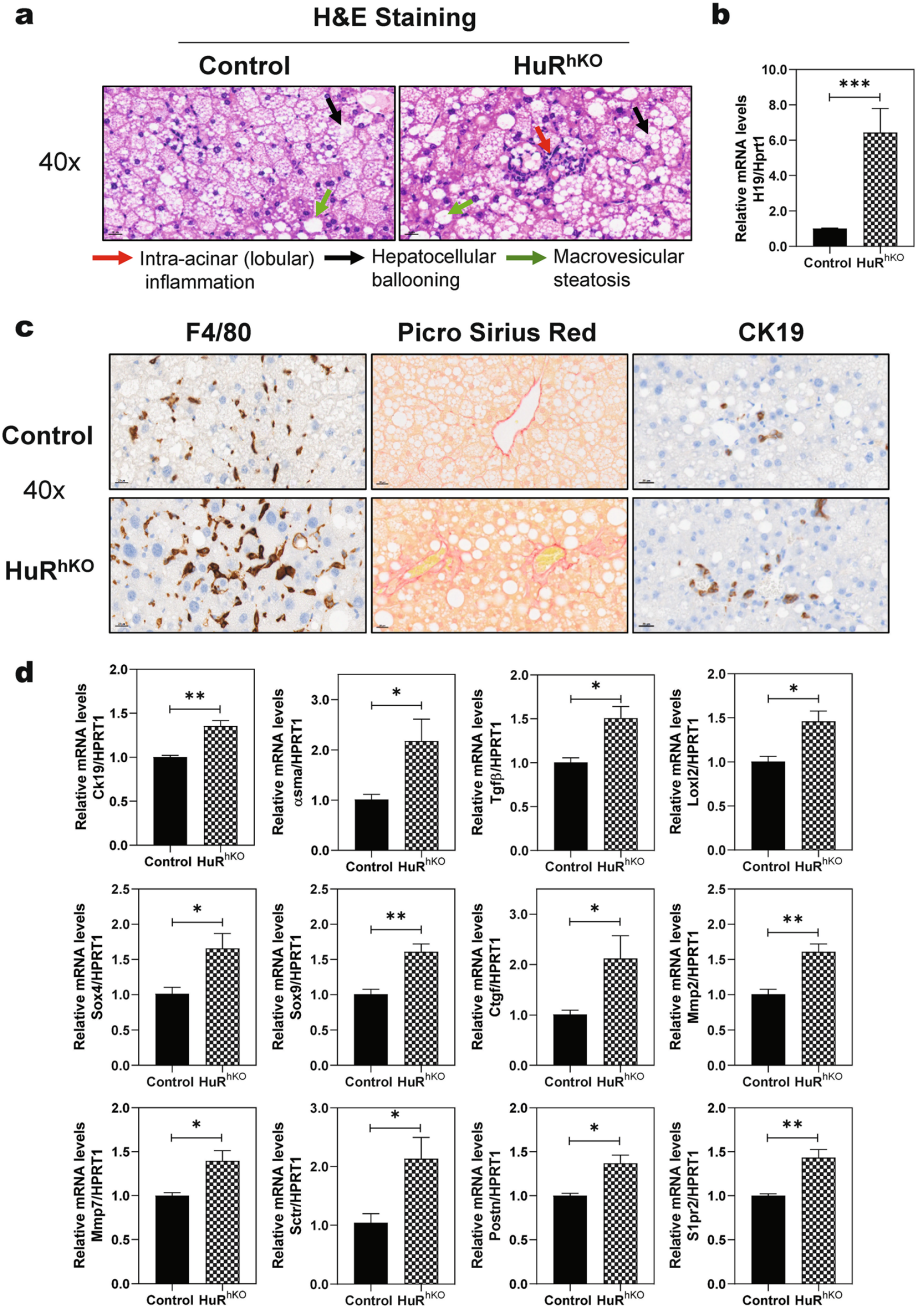
Hepatocyte-specific HuR deficiency aggravates WDSW-induced hepatic fibrosis. HuR^hKO^ and control mice were fed ad libitum a WDSW for 12 weeks. **a** Representative H&E staining images of the liver sections (scale bar, 20 μm for 40× magnification). **b** Relative mRNA levels of H19 in the liver of HuR^hKO^ and control mice. **c** Representative images of the liver sections stained with F4/80, Picro Sirius Red staining, and IHC staining of CK19 (scale bar, 20 μm for 40 × magnification). **d** Relative mRNA levels of genes involved in hepatic fibrosis were determined by real-time RT–PCR and normalized with HPRT1 as an internal control. Relative mRNA levels of Ck19, α-Sma, Tgfβ, Lox12, Sox4, Sox9, Ctgf, Mmp2, Mmp7, Sctr, Postn, and S1pr2 are shown. Data are expressed as the mean ± SEM. Statistical significance relative to control: *p < 0.05, **p < 0.01, ***p < 0.001, n = 10–12.

To further examine the impact of hepatic HuR in WDSW-induced NAFLD disease progression, both control and HuR^hKO^ mice were fed ad libitum a WDSW for 12 weeks to induce NASH and early fibrosis. As shown in Additional file 1: Fig. S10a, HuR protein levels were significantly downregulated in the livers of HuR^hKO^ mice. As expected, H&E staining showed that HuR^hKO^ mice fed WDSW for 12 weeks exacerbated intra-acinar (lobular) inflammation, hepatocellular ballooning, and macrovesicular steatosis (Fig. 7a). Similar to the 4-week WDSW feeding study in HuR^hKO^ mice (Fig. 4d), the hepatic H19 expression level was significantly upregulated in 12-week WDSW-feeding HuR^hKO^ mice (Fig. 7b). The key genes involved in the fatty acid synthesis and lipid metabolism were also increased in HuR^hKO^ mice compared to the control mice, including sterol regulatory element-binding protein 2 (Srebp2), Pparα, elovl fatty acid elongase 7 (Elovl7), 3-hydroxy-3-methylglutaryl coenzyme-A (HMG-CoA) reductase (HMG-CoAR), PPARG Coactivator 1 Alpha (Pgc1α), lipoprotein lipase (Lpl), fibroblast growth factor 21 (Fgf21), fatty acid desaturase (Fads2), and Cyclin D1 (Additional file 1: Fig. S10b). As shown in Fig. 7c, HuR^hKO^ mice fed WDSW for 12 weeks resulted in enhanced macrophage infiltration to the liver, as indicated by IHC staining of F4/80. In addition, the mRNA levels of F4/80, Cd11b, Cd63, Cd68, Ccl2, Ccr2, Tnfα, Cd14, Tlr4, ceramide kinase (Cerk), Caspase 1, and cyclooxygenase 2 (Cox-2) were significantly increased in the liver of HuR^hKO^ mice (Additional file 1: Fig. S11a). Furthermore, as shown in Additional file 1: Fig. S11b, the expression levels of key genes involved in neutrophil activation were also significantly upregulated in the liver of HuR^hKO^ mice fed WDSW for 12 weeks, including Nox2, neutrophil cytosolic factor 1 (Ncf1), Ncf2, Ncf4, Cybα, Il2rg, Elastin, and Selectin. These findings were consistent with the results in HuR^hKO^ mice fed WDSW for 4 weeks, indicating hepatocyte-specific HuR deficiency enhances WDSW-induced dysregulation of hepatic lipid metabolism and activation of inflammatory response.

Hepatic cell injury and inflammation are the major driving forces of hepatic fibrosis, which is closely associated with mortality in NASH patients [37]. To determine the impact of hepatic HuR on the progression of NASH fibrosis, we performed Picro Sirus Red staining, IHC staining of CK-19 and real-time PCR analysis. As shown in Fig. 7c, 12-week WDSW-feeding induced early fibrosis in HuR^hKO^ mice but much less in control mice. CK-19 staining indicated that hepatocyte-specific HuR deficiency significantly exacerbated WDSW-induced cholangiocyte proliferation. The mRNA expression levels of fibrotic genes were significantly upregulated in 12-week WDSW-feeding HuR^hKO^ mice, including Ck19, smooth muscle actin (α-Sma), transforming growth factor-beta 1 (Tgfβ1), lysyl oxidase-like 2 (Loxl2), SRY (sex-determining region Y)-box 4&9 (Sox4&9), connective tissue growth factor (Ctgf), matrix metallopeptidase 2 and 7 (Mmp2&7), secretin receptor (Sctr), periostin (Postn), and S1pr2, indicating hepatocyte-specific HuR deficiency aggravates WDSW-induced hepatic fibrosis (Fig. 7d).

### H19 downregulation alleviates WDSW-induced NAFLD in HuR^hKO^ mice

**Fig. 8:**
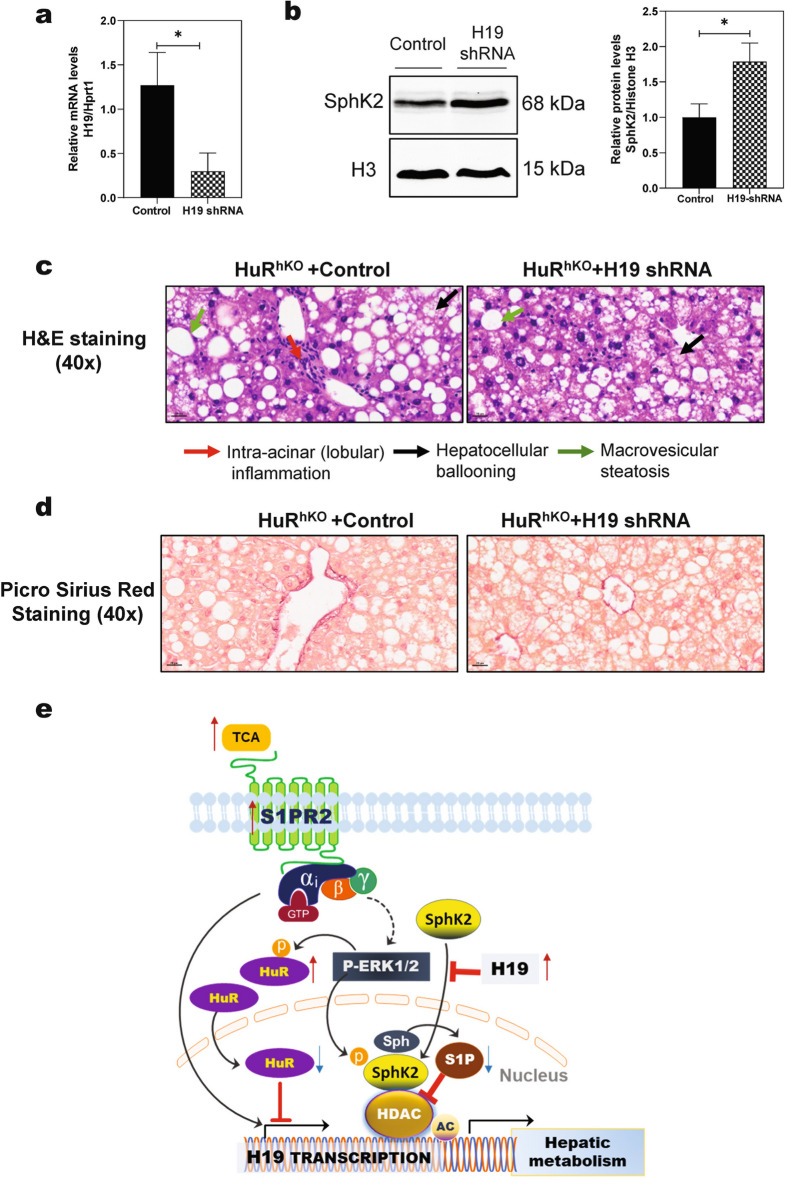
H19 downregulation alleviates WDSW-induced NAFLD in HuR^hKO^ mice. HuR^hKO^ mice were injected with a recombinant adenovirus encoding an H19 shRNA or control adenovirus (GFP) while being fed ad libitum a WDSW for 4 weeks. **a** Relative mRNA levels of H19 in livers injected with H19 shRNA adenovirus compared control adenovirus. Data are expressed as the mean ± SEM. Statistical significance relative to control: *p < 0.05, n = 5. **b** Representative immunoblot images of nuclear SphK2 in the liver are shown. Relative protein levels of nuclear SphK2 were normalized using histone H3. Statistical significance relative to control: *p < 0.05, n = 5. **c** Representative H&E staining images of the liver sections (scale bar, 20 μm for 40 × magnification). **d** Representative Picro Sirius Red staining images of the liver sections (scale bar, 20 μm for 40 × magnification). **e** Schematic diagram of the potential mechanism of HuR in attenuating WDSW-induced NAFLD. The current study demonstrated that HuR functions as an important regulator of hepatic lipid metabolism, enterohepatic bile acid homeostasis, inflammation, and fibrosis by suppressing H19 expression and modulating the SphK2 nuclear protein level. Bile acid-induced activation of S1PR2 may also contribute to NASH fibrosis. Hepatocyte-specific modulation of HuR expression and its downstream target, H19, may be used to develop potential therapeutics for NAFLD

Based on our results and published RNAseq data, H19 upregulation may represent a major cellular mechanism underlying WDSW-induced NAFLD in HuR^hKO^ mice. To verify the role of H19 in WDSW-induced NAFLD in HuR^hKO^ mice, HuR^hKO^ mice were injected with a recombinant adenovirus encoding an H19 shRNA or control adenovirus (GFP) while being fed ad libitum a WDSW for 4 weeks. As shown in Fig. 8a, the H19 levels in the liver were significantly decreased after injection of adenovirus of H19 shRNA compared to injection of control adenovirus. Down-regulation of H19 reversed the WDSW-induced decrease of nuclear SphK2 protein (Fig. 8b). H&E staining showed that downregulation of H19 in HuR^hKO^ mice reduced WDSW-induced intra-acinar (lobular) inflammation, hepatocellular ballooning, and macrovesicular steatosis (Fig. 8c). The Picro-Sirius Red staining also showed that downregulation of H19 in HuR^hKO^ mice reduced WDSW-induced early fibrosis (Fig. 8d). Together, these results suggest that the upregulation of H19, at least partially, contributes to WDSW-induced NAFLD development in HuR^hKO^ mice.

## Discussion

The prevalence of NAFLD has been continuously increasing during the last decade globally due to obesity. Recently, there has been compelling evidence supporting the “Multi-hit” over the “Two-hit” hypothesis of NAFLD pathogenesis [2, 38–40]. In addition to the dysregulation of hepatic lipid metabolism, the progression from NAFL to NASH is correlated with systemic and adipose tissue inflammation, individual genetic and epigenetic factors, complex environmental factors, dysbiosis and disruption of bile acids homeostasis [2, 40]. Histologically, NASH is characterized by steatosis, inflammation, hepatocyte injury (ballooning), and/or fibrosis [40]. Emerging evidence has demonstrated the involvement of HuR in the pathogenesis of various liver diseases, including fatty liver diseases, hepatic inflammation, viral hepatitis, liver fibrosis, and liver cancers [20–22, 41]. In the present study, we demonstrated that in HuR^hKO^ mice, lack of HuR in hepatocytes exacerbated the progression of WDSW-induced NAFLD as indicated by enhanced liver steatosis, inflammation, and fibrosis, consistent with the findings in the recent studies [20]. Using RNA transcriptome analysis combined with histological examination, we found that hepatocyte-specific HuR deficiency upregulated the expression of H19 and S1PR2 (Figs. 4, 5). Our current study also discovered that hepatocyte-specific HuR deficiency significantly disrupted bile acid homeostasis.

Two studies reported that HuR played a critical role in modulating lipid homeostasis in response to metabolic stress using liver-specific HuR knockout mice [20, 41]. In the study by Zhang et al. no significant change was noticed in hepatic lipid accumulation and liver function under a chow diet [41]. However, the recent study by Subramanian, P. et al. showed significant hepatic lipid accumulation in liver-specific HuR knockout mice under normal diet feeding [20]. Since studies did not specify the gender and age of the mice, the discrepancy could be due to the gender and age difference or other environmental factors. In our HuR^hKO^ mice, we did not observe a significant change in hepatic lipid accumulation under a normal diet condition (Additional file 1: Fig.S1j).

Hepatic lipid accumulation is associated with dysregulated fatty acid biosynthesis and lipid metabolism [42]. The results of the current study showed that the essential genes involved in fatty acid biosynthesis were significantly increased in the liver of HuR^hKO^ mice fed WDSW for 4 or 12 weeks (Fig. 2 and Additional file 1: Fig. S10). Inflammation and oxidative stress are important drivers for NAFLD disease progression [27, 43, 44]. Our data showed that hepatocyte-specific deficiency of HuR significantly increased the expression of macrophage markers, various chemokines, innate immune responses markers, and chemokines in the livers of HuR^hKO^ mice (Fig. 3 and Additional file 1: Fig. S11a). The activation of neutrophils contributes to NAFLD/NASH progression [45, 46]. We have previously reported that neutrophils were activated in a WDSW-induced NASH mouse model [24]. Consistently, RNA-seq data and real-time RT–PCR results indicated that hepatocyte-specific deficiency of HuR was able to exacerbate WDSW-induced activation of neutrophils (Fig. 3d and Additional file 1: Fig. S3b and S11b). Hepatic fibrosis is a vital marker during the progression from NAFL to NASH. The results of our current study demonstrated that hepatic deficiency of HuR also induced early hepatic fibrosis by modulating key genes involved in hepatic stellate cell activation and cholangiocyte proliferation after a WDSW feeding for 12 weeks (Fig. 7c, d). In contrast, a previous study reported that HuR is upregulated in the liver of human cirrhotic patients and BDL mouse models. HuR silencing using siRNA had beneficial functions during BDL-induced cholestatic liver injury and HSC activation [47]. However, the cell-type-specific role of HuR in this study remains unclear. Based on our current data, it is clear that HuR may play a protective role in hepatocytes in the progression of NAFL to NASH. Consistent with this hypothesis, we found that HuR protein level was significantly reduced in NASH–HCC patients (Additional file 1: Fig. S12a, b).

H19 is the first lncRNA identified and characterized as the first imprinted gene in eukaryotes as a hepatic fetal-specific non-translatable mRNA in the late 1980s and has been implicated in various liver diseases [12]. We have previously reported that H19 promoted hepatic stellate cell activation and cholestatic liver fibrosis [13, 14]. The current studies showed that H19 was highly expressed in human NASH patients and NASH mouse models (Figs. 4a, b). We also found that hepatic H19 level was significantly upregulated in HuR^hKO^ mice fed-WDSW for 4 or 12 weeks (Figs. 4d and 7b). Interestingly, in a recent study with liver specific HuR knockout mice, H19 is the most significantly upregulated gene (Fig. 4c, GSE143358). Similarly, in a new NASH–HCC mouse model, H19 ranked the top ninth among significantly upregulated genes (Fig. 4c, GSE197884). We further showed that HuR inhibited H19 transcription by suppressing the H19 promoter activity (Fig. 4e). Although HuR has been extensively studied as an RNA-binding protein, it has been reported that many RNA-binding proteins, including HuR, can function as transcription factors [48]. Furthermore, H19 deficiency protected mice from WDSW-induced NAFLD (Fig. 4f); and downregulation of H19 in HuR^hKO^ mice significantly reduced WDSW-induced hepatic lipid accumulation, inflammation and liver fibrosis (Fig. 8c, d).

We and others have previously reported that SphK2 is an important regulator of hepatic lipid metabolism and ER stress [9, 10]. Consistently, in the current study, we also found that the nuclear SphK2 levels were dramatically decreased in the liver of WDSW-induced HuR^hKO^ mice but increased in WDSW-induced H19^−/−^ mice (Fig. 5a, b). However, neither HuR nor H19 had significant effects on the total protein level of SphK2 (Additional file 1, Fig.S6). The results indicated that HuR or H19 might regulate the translocation of SphK2 from the cytosol into the nucleus. Consistently, downregulation of H19 using an H19 shRNA in HuR^hKO^ mice increased the nuclear protein level of SphK2 in the liver (Fig. 8b).

Bile acids are important signaling molecules in regulating hepatic lipid metabolism, inflammation, and fibrotic response. Altered circulating bile acid composition is associated with the severity of NASH [23]. Increased hepatic/serum conjugated primary bile acids may be an important factor in cholestatic liver injury and liver fibrosis by activating S1PR2 and H19 [13, 33]. Consistent with the previous studies, total conjugated primary bile acids were significantly increased in the serum of WDSW-fed HuR^hKO^ mice, together with the increased total bile acids and TCA (Fig. 1d, e, Additional file 1: Table S2-3). In addition, the current study showed that hepatic deficiency of HuR disrupted bile acid homeostasis by modulating key enzymes of bile acid synthesis, nuclear receptors, and hepatic bile acid transporters (Fig. 6a). Interestingly, the β-MCA level was significantly decreased in the serum and liver of WDSW-fed HuR^hKO^ mice (Fig. 1d and 6b). This may be caused by the down-regulation of Cyp2c70 (Additional file 1: Fig. S12c), which catalyzes the conversion of CDCA into αMCA and possible reduction of gut microbiome-mediated epimerization of 7α-MCA into 7β-MCA [49]. In addition, hepatocyte-specific deficiency of HuR induced dysregulation of intrahepatic bile acid homeostasis under both normal diet and WDSW feeding conditions (Fig. 6b–d and Additional file 1: Figs.S7–S9, and Additional file 1: Tables S4–S17). These findings provided strong evidence for a protective role of hepatocyte-specific HuR in NAFLD progression by modulating enterohepatic bile acid circulation.

We have previously reported that TCA-induced activation of S1PR2 promotes cholestatic liver injury [33]. In the current study, we also found that S1PR2 expression was upregulated in the livers of human NASH patients and NASH mice (Fig. 5c). Importantly, we found both mRNA and protein levels of S1PR2 were upregulated in HuR^hKO^ mice (Fig. 5c, d). Moreover, S1PR2 overexpression enhanced H19 transcription by increasing the H19 promoter activity (Fig. 5e). Since S1PR2 is a cell membrane GPCR, its effect on the H19 promoter is likely mediated by its downstream signaling molecules via activation of ERK1/2. Although the current study showed that S1PR2 expression was increased in HuR^hKO^ mice fed with WDSW, the luciferase reporter assay indicated that HuR had no direct effect on S1PR2 expression (Data not shown). Our previous studies showed that conjugated bile acids activate ERK via the S1PR2, resulting in the activation of nuclear SphK2 [10, 36]. Activation of SphK2 ameliorates metabolic stress-induced hepatic lipid accumulation [9]. In HuR^hKO^ mice, upregulation of H19 reduced SphK2 nuclear translocation under WDSW feeding condition.

## Conclusion

In summary, as illustrated in Fig. 8e, the current study has demonstrated that HuR functions as an important regulator of hepatic lipid metabolism, enterohepatic bile acid homeostasis, inflammation, and fibrosis by suppressing H19 expression and modulating SphK2 nuclear protein level. Bile acid-induced activation of S1PR2 may also contribute to NASH fibrosis via upregulating H19. In addition, the phosphorylation status of HuR impacts its intracellular localization. It has been reported that ERK-mediated phosphorylation of HuR increases cytosolic HuR levels and reduces nuclear HuR [50, 51]. Hepatocyte-specific modulation of HuR expression and its downstream target, H19, may be used to develop potential therapeutic targets for NAFLD.

## Materials and methods

### Animal experiments

Both male and female mice were used for all in vivo studies. *HuR*^*flox/flox*^ mice (C57BL/6 J) were from the Jackson Laboratory (Bar Harbor, ME). Hepatocyte-specific HuR knockout (HuR^hKO^) mice were generated by tail-vein injection of *HuR*^*flox/flox*^ mice with AAV8-thyroxine-binding globulin promoter (TBGP)-Cre recombinase from Addgene (Watertown, MA). Control mice were injected with the same amount of AAV8-TBGP-GFP from Vector Biolabs (Malvern, PA). Age and gender-matched HuR^hKO^ and control mice were fed ad libitum a Western diet, with 42% kcal from fat and containing 0.1% cholesterol from Envigo (Cat#: TD.88137, Indianapolis, IN) with a high fructose-glucose solution (23.1 g/L d-fructose + 18.9 g/L d-glucose) (Western diet plus sugar water, WDSW) for 4 weeks or 12 weeks. Age and gender-matched H19 knockout (H19^−/−^) (the maternal H19ΔExon1/ + mice with C57BL/6 J background generated by Dr. Karl Pfeifer at NIH and provided by Dr. Jian-Ying Wang at the University of Maryland (Baltimore, MD, USA) and WT mice were fed ad libitum a WDSW for 4 weeks. An adenovirus expressing H19 shRNA (A gift from Dr. Li Wang, The Institute for Systems Genomics at the University of Connecticut) was used to knock down H19 expression in HuR^hKO^ mice via tail-vein injection and adenovirus of GFP was used as a control. All mice were housed in a 12 h light/12 h dark cycle with a controlled room temperature between 21 and 23 °C and free access to water. All animal experiments were performed following institutional guidelines for ethical animal studies and approved by the VCU Institutional Animal Care and Use Committee. At the end of the experiment, mice were weighed and anesthetized by exposure to inhaled isoflurane. Blood was collected by cardiac puncture. The serum was collected and stored at − 80 °C for later analysis. After euthanasia, the liver was collected for histological analysis, RNA profiling, and Western blot analysis.

### Transient transfection and dual-luciferase report assay

HEK-293 cells with a density of 75% were inoculated into 24-well plates at a density of 2 × 10^5^ cells per well and cultured for 24 h. The cells were transfected with pGL3-human H19-promoter (a gift from Dr. Xiaodi Tan at Northwestern University (Chicago, IL) or pGL3-control vector together with pcDNA3-TAP-human HuR or pcDNA3-TAP vector (gifts from Dr. Jian-Ying Wang at University of Maryland) along with Renilla control vector, pGL4.76-hRluc/Hygro from Promega (Madison, WI) using PolyJet from SignaGen Laboratories (Frederick MD) according to the manufacturer's instructions (n = 6). After 48 h, the cells were washed with PBS, and the cell lysates were collected for firefly and Renilla luciferase activity assays using a dual-luciferase reporter assay kit (E1910) from Promega (Madison, WI). The promoter activity was expressed using the ratio of firefly luciferase activity to Renilla luciferase activity.

### Human liver tissues

Frozen human liver tissues (Healthy and NASH patients, both male and female) were obtained through the Liver Tissue Cell Distribution System (Minneapolis, MN), funded by the National Institutes of Health (Contract# HSN276201200017C).

### Statistical analysis

Data are expressed as the mean ± SEM from at least three independent experiments. The student's t-test was used to analyze the difference between the two groups by GraphPad Prism (version 8; GraphPad Software Inc., San Diego, CA). p ≤ 0.05 was considered statistically significant.

## Supplementary Information

**Additional file 1: **Additional methods, Supplementary figures, Supplementary tables, and raw image files. The accession number for the raw data FASTQ and processed data file deposit in NCBI is GEO: GSE231215.

## Data Availability

Detailed methods and datasets generated and/or analyzed during the current study are available in the Additional file 1.

## Abbreviations

NAFLD: Nonalcoholic fatty liver disease
HuR: Human antigen R
HuR^hKO^: Hepatocyte-specific HuR knockout
WDSW: Western diet plus sugar water
SphK2: Sphingosine kinase 2
NASH: Nonalcoholic steatohepatitis
HCC: Hepatocellular carcinoma
S1PR2: Sphingosine-1 phosphate receptor 2
lncRNA: Long noncoding RNA
H19^−^^/^^−^: H19 knockout
RNA-Seq: RNA Sequencing
BA: Bile acid
H&E: Hematoxylin and eosin
IHC: Immunohistochemistry
TCA: Taurocholic acid
Fads: Fatty acid desaturases
Lxr: Liver X receptor
Ppar: Peroxisome proliferator-activated receptor
