# Supplementary material for: RNA binding protein HuR protects against NAFLD by suppressing long noncoding RNA H19 expression
Source: Cell Biosci. 2022 Oct 12;12:172. doi: 10.1186/s13578-022-00910-7 (PMC9558407; doi:10.1186/s13578-022-00910-7)

Figure S1

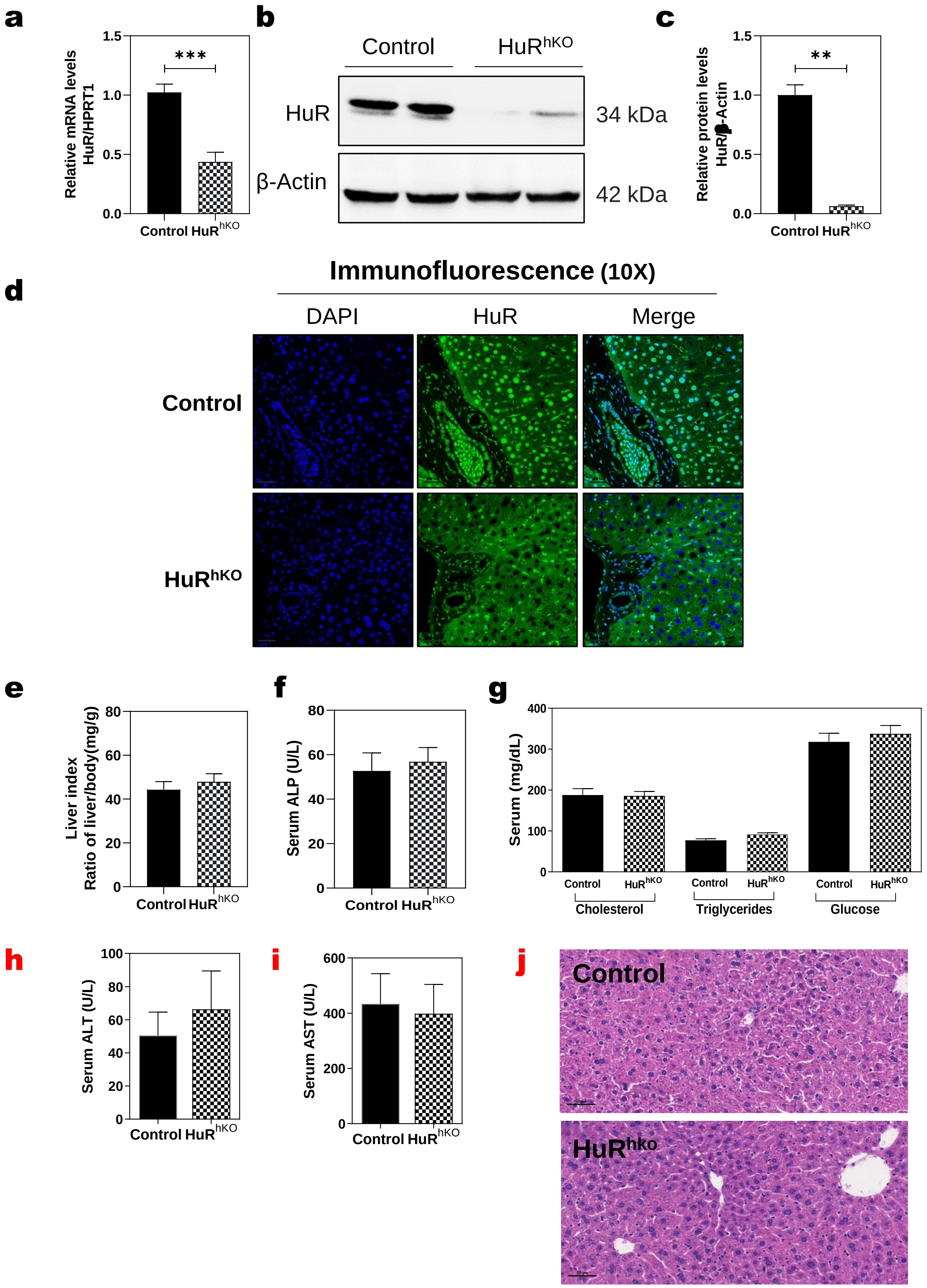
a
Relative mRNA levels
HuR/HPRT1
1.5
1.0
0.5
0.0
***
Control HuR^hKO
b
Control
HuR^hKO
HuR
β-Actin
34 kDa
42 kDa
c
Relative protein levels
HuR/β-Actin
1.5
1.0
0.5
0.0
**
Control HuR^hKO
d
Immunofluorescence (10X)
DAPI
HuR
Merge
Control
HuR^hKO
e
Liver index
Ratio of liver/body(mg/g)
80
60
40
20
0
Control HuR^hKO
f
Serum ALP (U/L)
80
60
40
20
0
Control HuR^hKO
g
Serum (mg/dL)
400
300
200
100
0
Control HuR^hKO Control HuR^hKO Control HuR^hKO
Cholesterol
Triglycerides
Glucose
h
Serum ALT (U/L)
100
80
60
40
20
0
Control HuR^hKO
i
Serum AST (U/L)
600
400
200
0
Control HuR^hKO
j
Control
HuR^hko

Figure S2

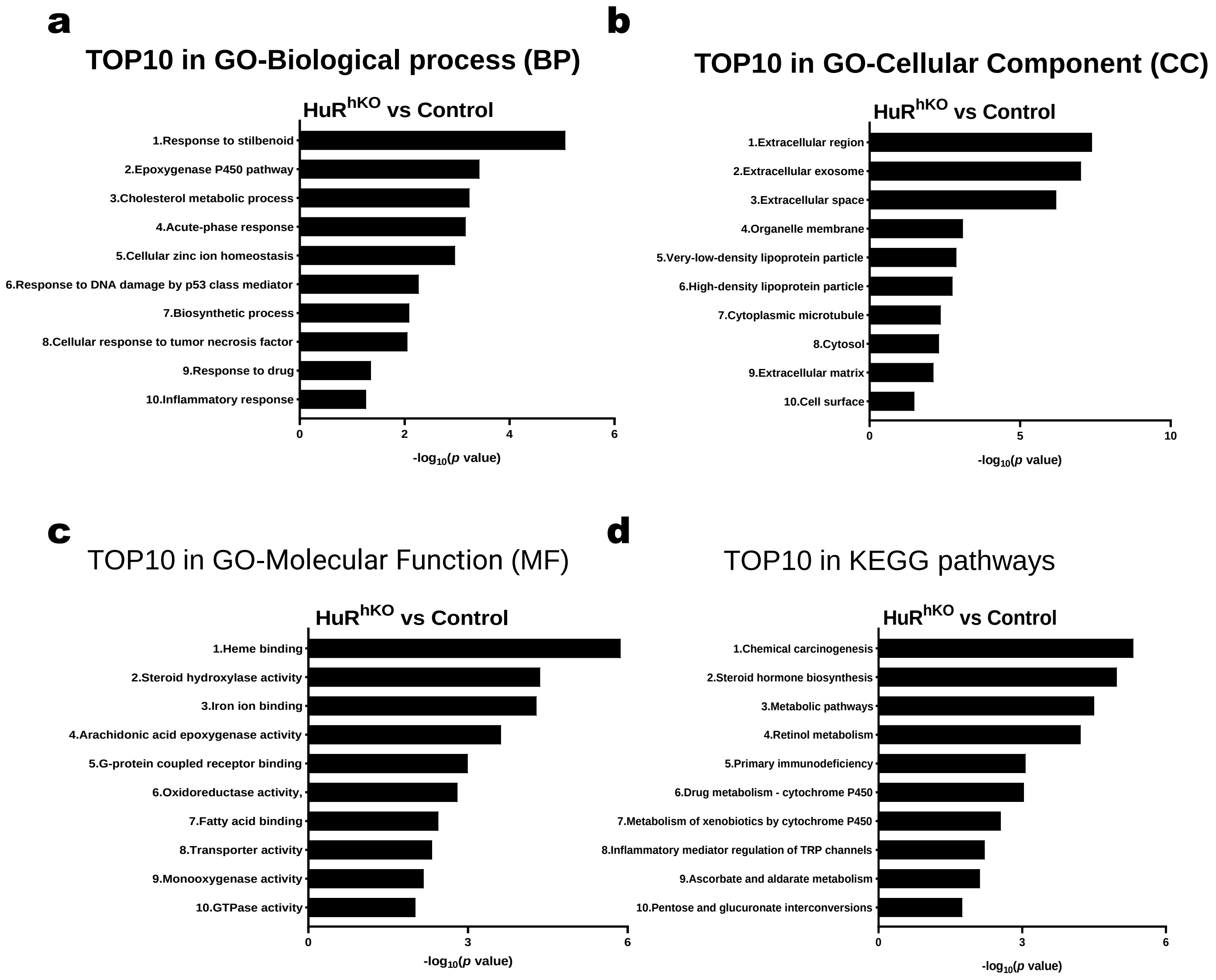
a
TOP10 in GO-Biological process (BP)
HuR^hKO vs Control
1.Response to stilbenoid
2.Epoxygenase P450 pathway
3.Cholesterol metabolic process
4.Acute-phase response
5.Cellular zinc ion homeostasis
6.Response to DNA damage by p53 class mediator
7.Biosynthetic process
8.Cellular response to tumor necrosis factor
9.Response to drug
10.Inflammatory response
0 2 4 6
-log10(p value)
b
TOP10 in GO-Cellular Component (CC)
HuR^hKO vs Control
1.Extracellular region
2.Extracellular exosome
3.Extracellular space
4.Organelle membrane
5.Very-low-density lipoprotein particle
6.High-density lipoprotein particle
7.Cytoplasmic microtubule
8.Cytosol
9.Extracellular matrix
10.Cell surface
0 5 10
-log10(p value)
c
TOP10 in GO-Molecular Function (MF)
HuR^hKO vs Control
1.Heme binding
2.Steroid hydroxylase activity
3.Iron ion binding
4.Arachidonic acid epoxygenase activity
5.G-protein coupled receptor binding
6.Oxidoreductase activity,
7.Fatty acid binding
8.Transporter activity
9.Monooxygenase activity
10.GTPase activity
0 3 6
-log10(p value)
d
TOP10 in KEGG pathways
HuR^hKO vs Control
1.Chemical carcinogenesis
2.Steroid hormone biosynthesis
3.Metabolic pathways
4.Retinol metabolism
5.Primary immunodeficiency
6.Drug metabolism - cytochrome P450
7.Metabolism of xenobiotics by cytochrome P450
8.Inflammatory mediator regulation of TRP channels
9.Ascorbate and aldarate metabolism
10.Pentose and glucuronate interconversions
0 3 6
-log10(p value)

Figure S3

a

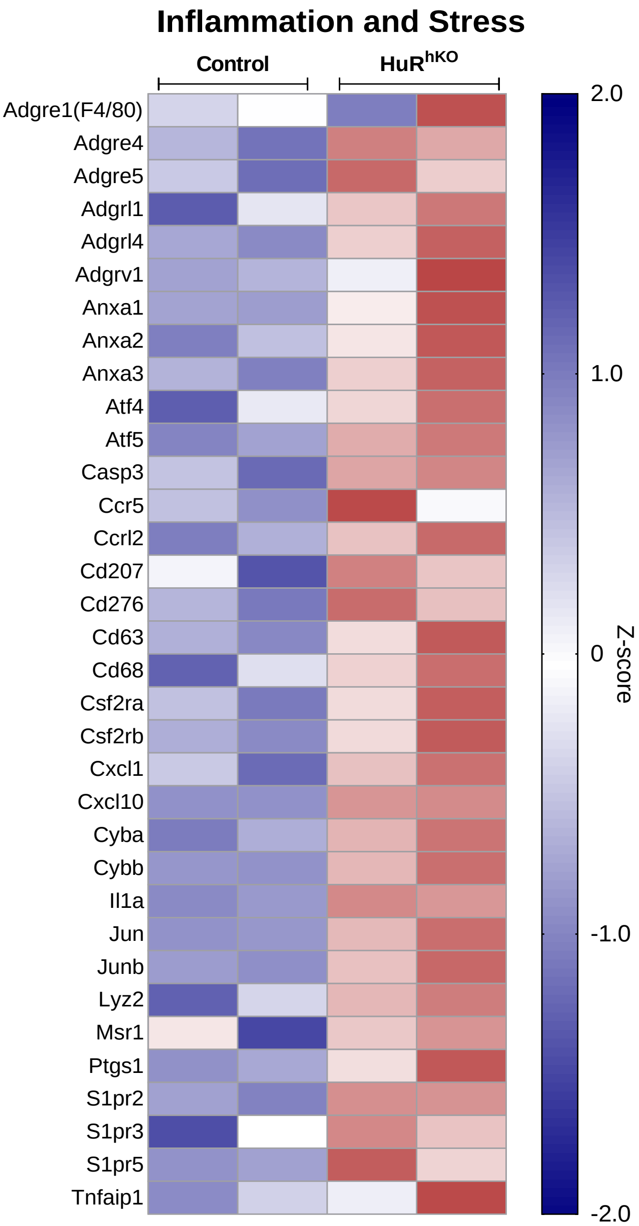
Inflammation and Stress
Control
HuR^{hKO}
Adgre1(F4/80)
Adgre4
Adgre5
Adgrl1
Adgrl4
Adgrv1
Anxa1
Anxa2
Anxa3
Atf4
Atf5
Casp3
Ccr5
Ccrl2
Cd207
Cd276
Cd63
Cd68
Csf2ra
Csf2rb
Cxcl1
Cxcl10
Cyba
Cybb
Il1a
Jun
Junb
Lyz2
Msr1
Ptgs1
S1pr2
S1pr3
S1pr5
Tnfaip1
2.0
1.0
0
-1.0
-2.0
Z-score

b

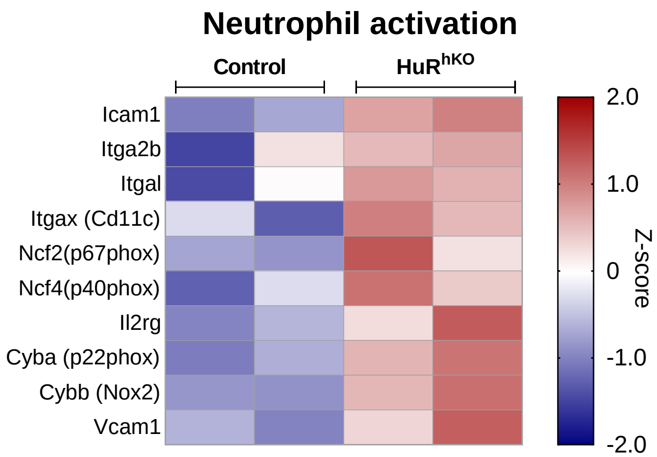
Neutrophil activation
Control
HuR^{hKO}
Icam1
Itga2b
Itgal
Itgax (Cd11c)
Ncf2(p67phox)
Ncf4(p40phox)
Il2rg
Cyba (p22phox)
Cybb (Nox2)
Vcam1
2.0
1.0
0
-1.0
-2.0
Z-score

Figure S4

Oxidative Phosphorylation

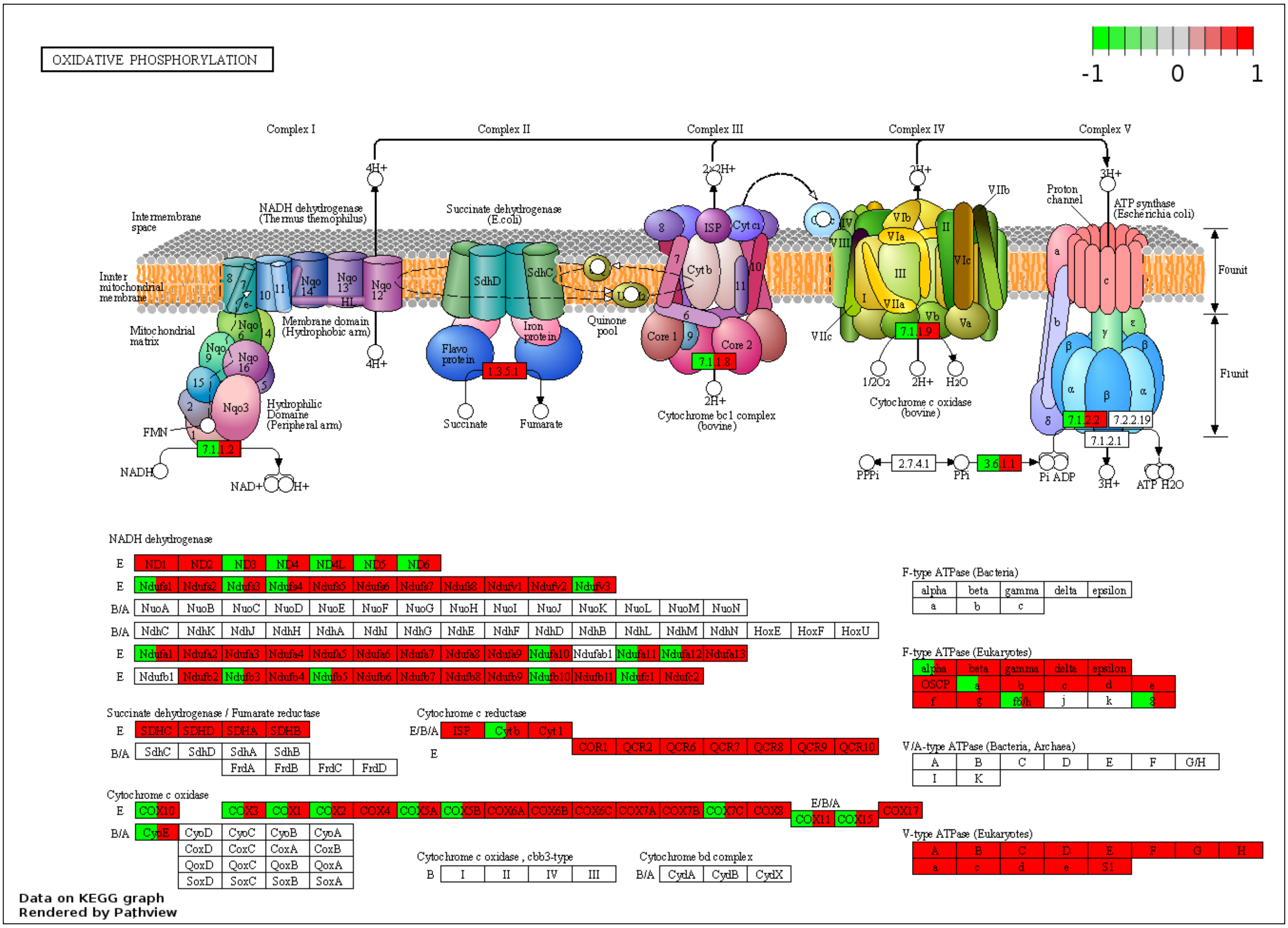

Figure S5

MAPK Signaling Pathway

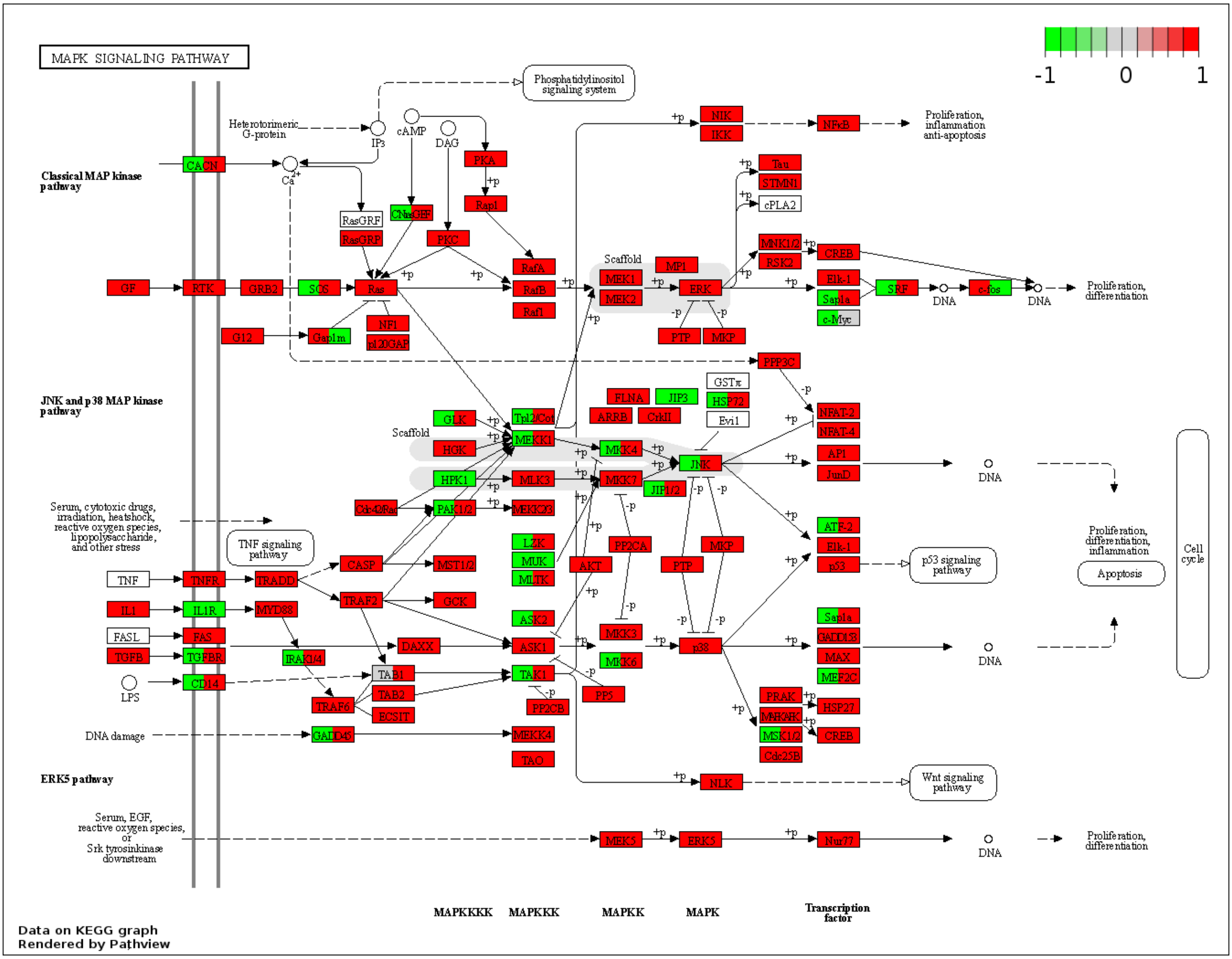

Figure S6

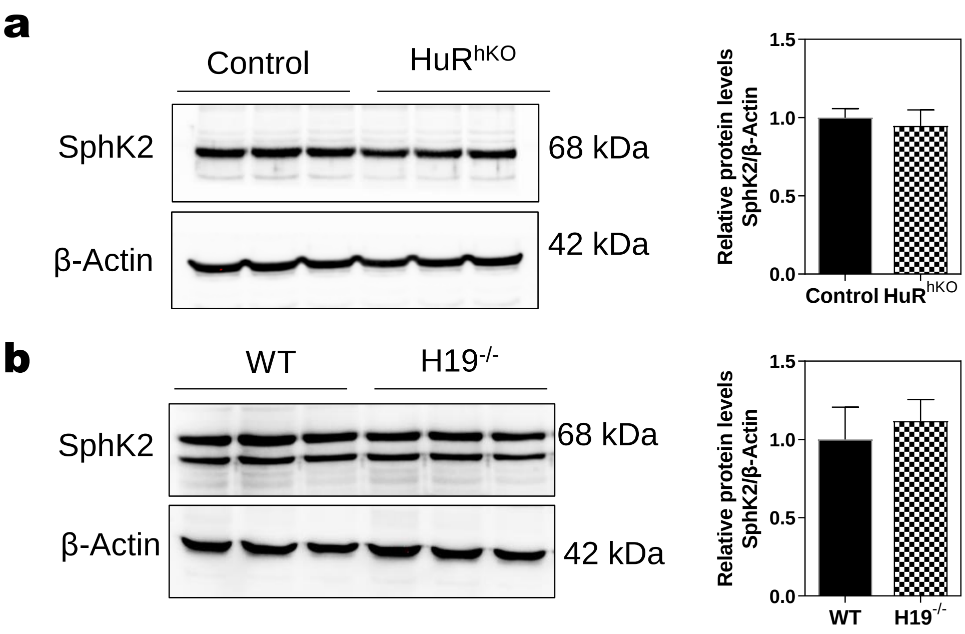
a
Control
HuR^hKO
SphK2
68 kDa
β-Actin
42 kDa
Relative protein levels SphK2/β-Actin
1.5
1.0
0.5
0.0
Control HuR^hKO
b
WT
H19^-/-
SphK2
68 kDa
β-Actin
42 kDa
Relative protein levels SphK2/β-Actin
1.5
1.0
0.5
0.0
WT
H19^-/-

Figure S7

a Bile acid composition in intestinal contents

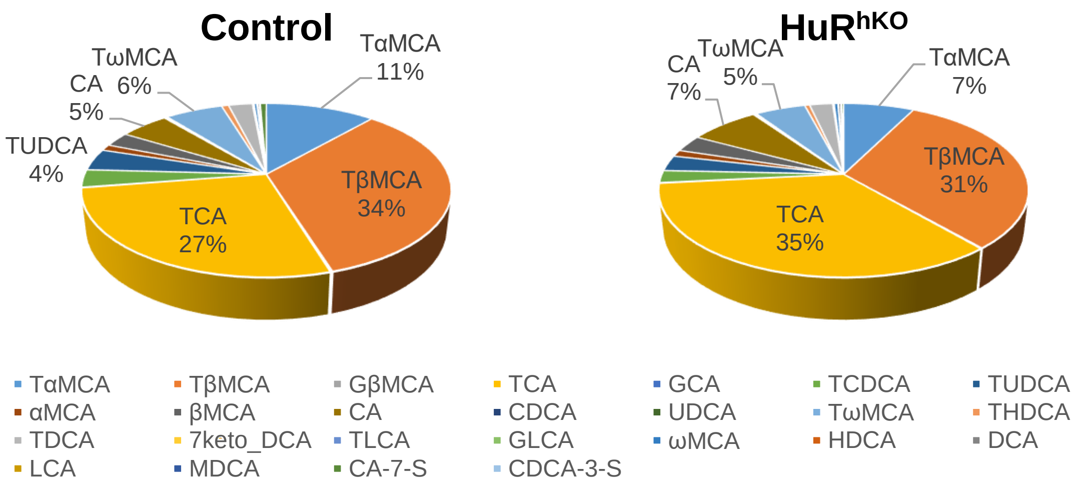

b Bile acids in intestinal contents

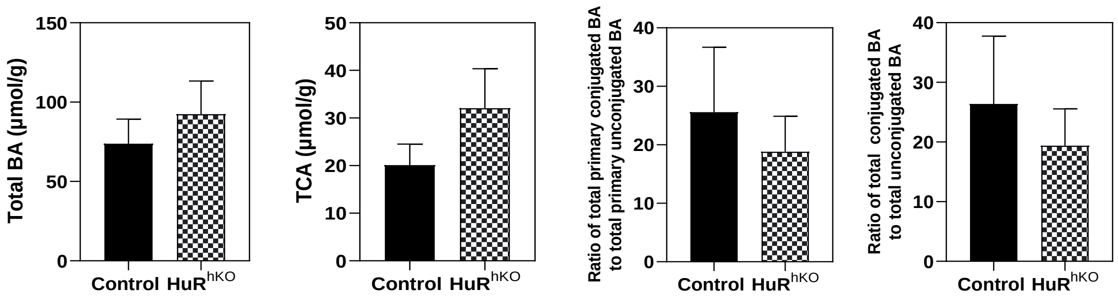

c Bile acid composition in cecal contents

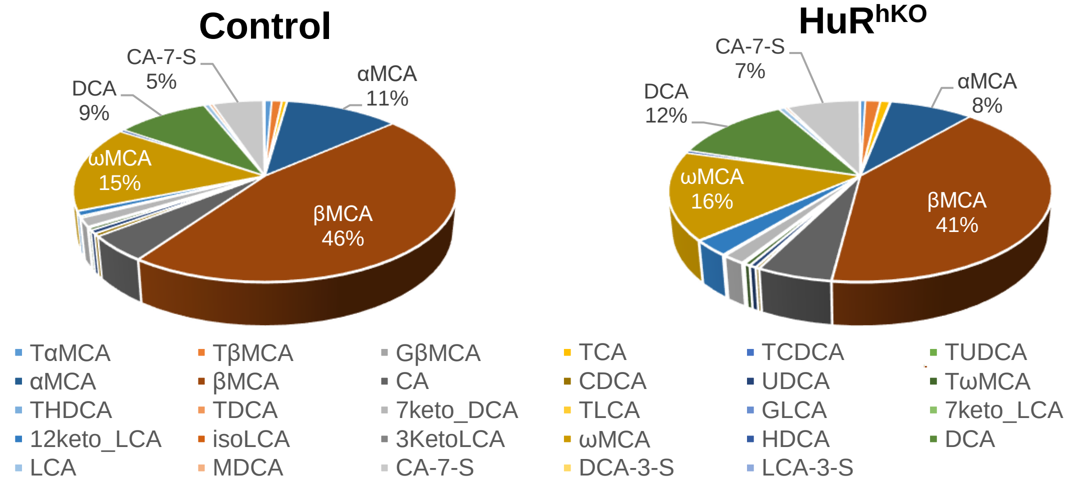

d Bile acids in cecal contents

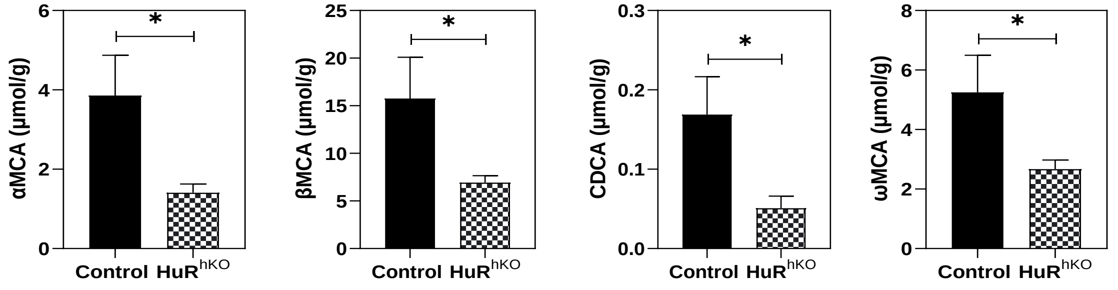

Figure S8

a **Bile acid composition in serum (normal diet)**

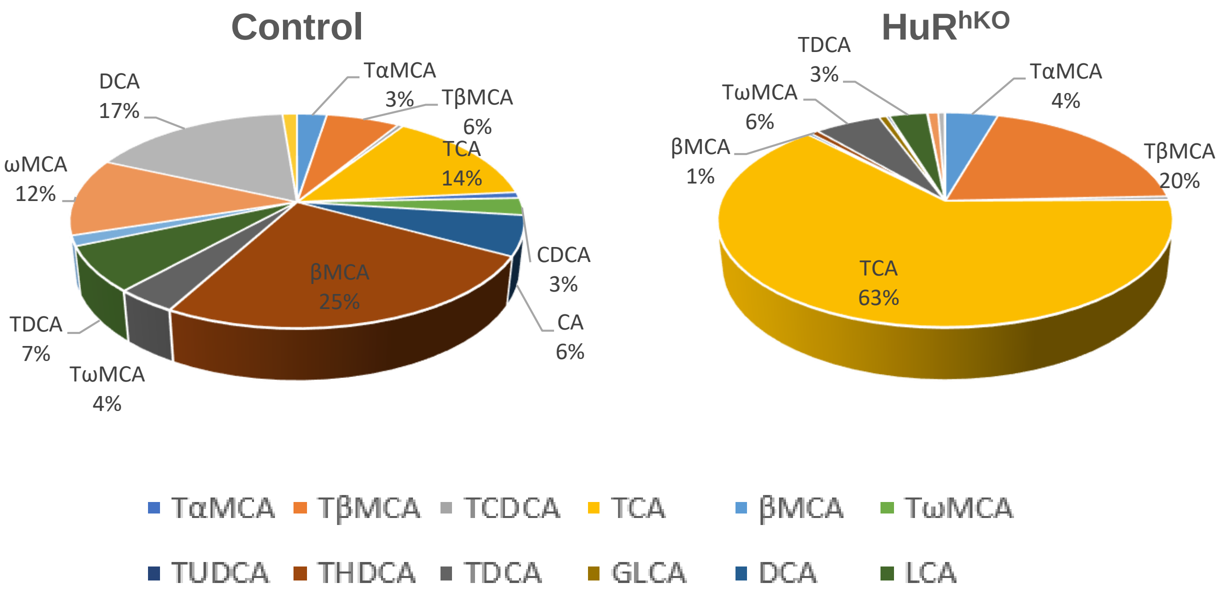

b **Bile acid composition in liver (normal diet)**

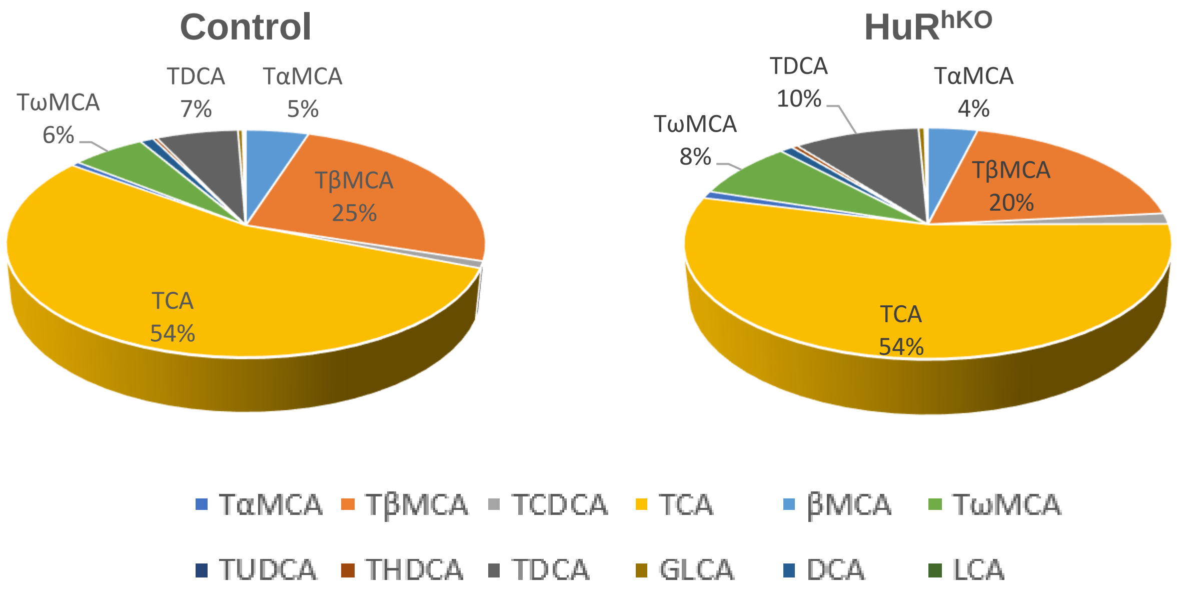

Figure S9

a Bile acid composition in intestinal contents (normal diet)

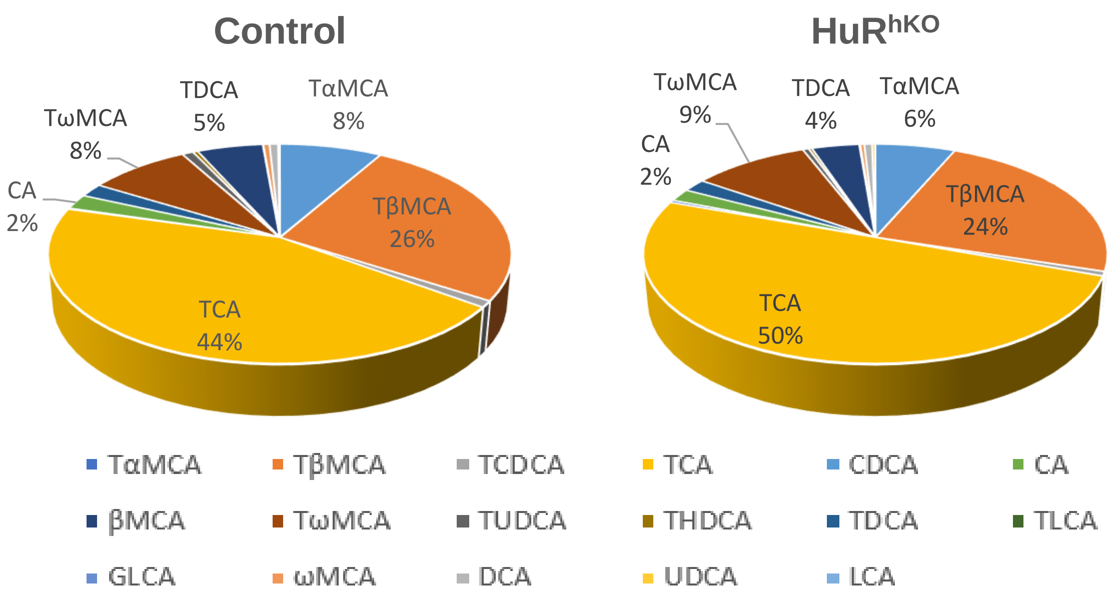

b Bile acid composition in cecal contents (normal diet)

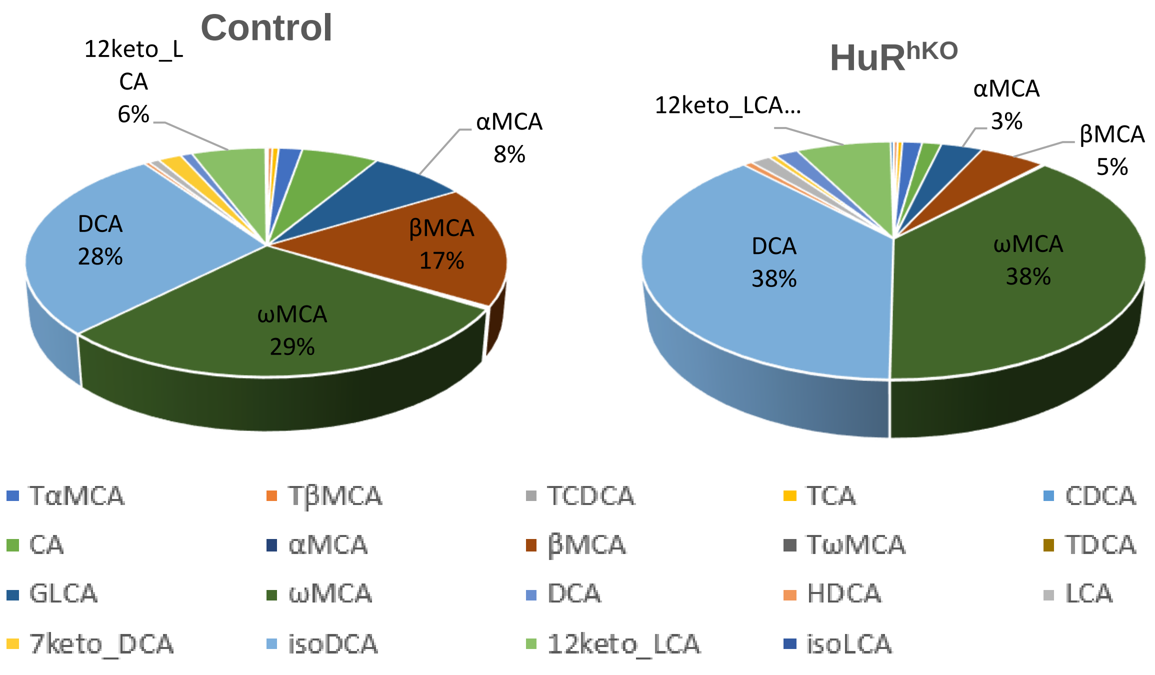

Figure S10

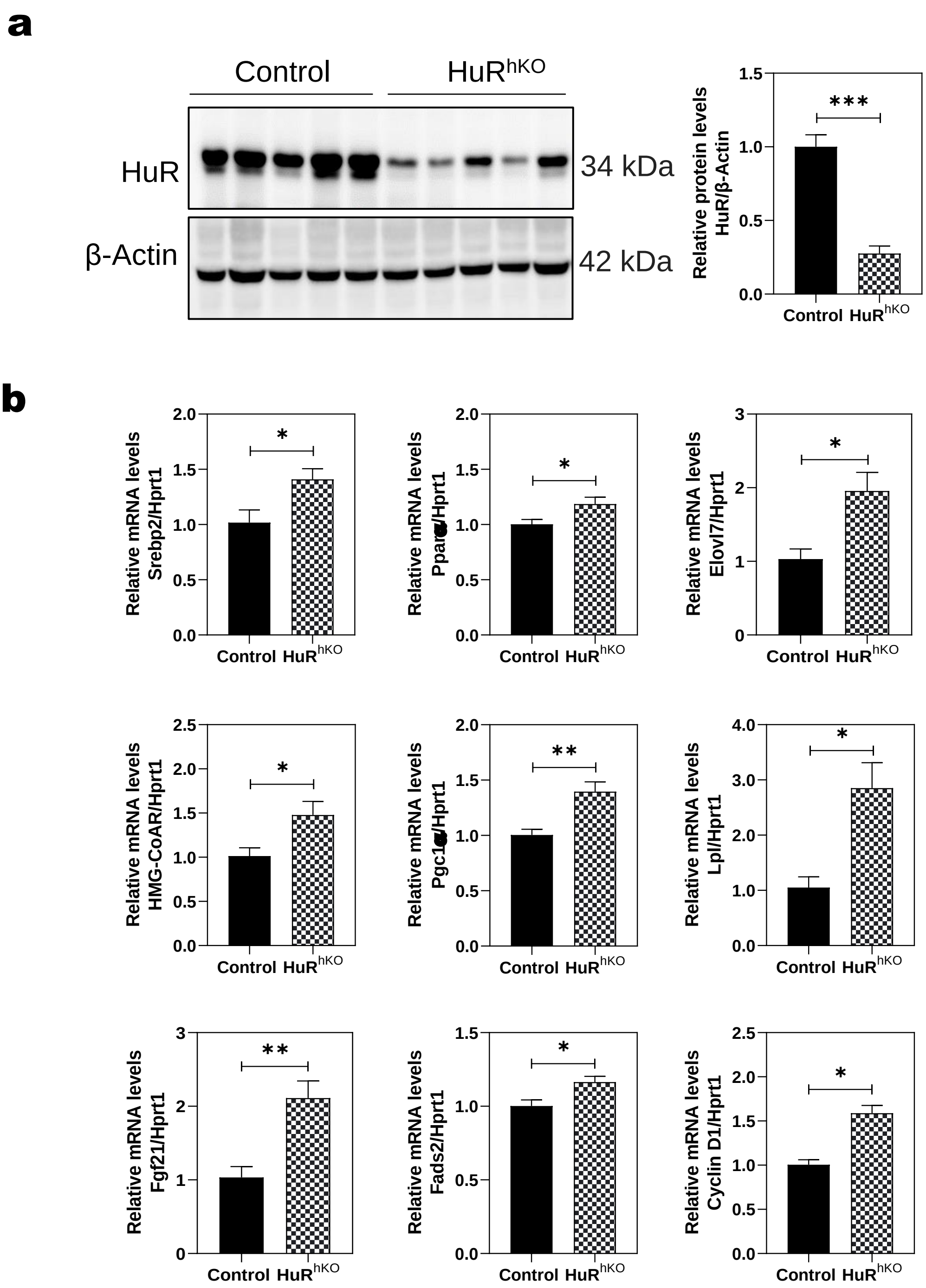
a
Control
HuR^hKO
HuR
β-Actin
34 kDa
42 kDa
Relative protein levels HuR/β-Actin
***
Control HuR^hKO
b
Relative mRNA levels Srebp2/Hprt1
Relative mRNA levels Elovl7/Hprt1
Relative mRNA levels HMG-CoAR/Hprt1
Relative mRNA levels Lpl/Hprt1
Relative mRNA levels Fgf21/Hprt1
Relative mRNA levels Fads2/Hprt1
Relative mRNA levels Cyclin D1/Hprt1
*
**
Control HuR^hKO

Figure S11

a

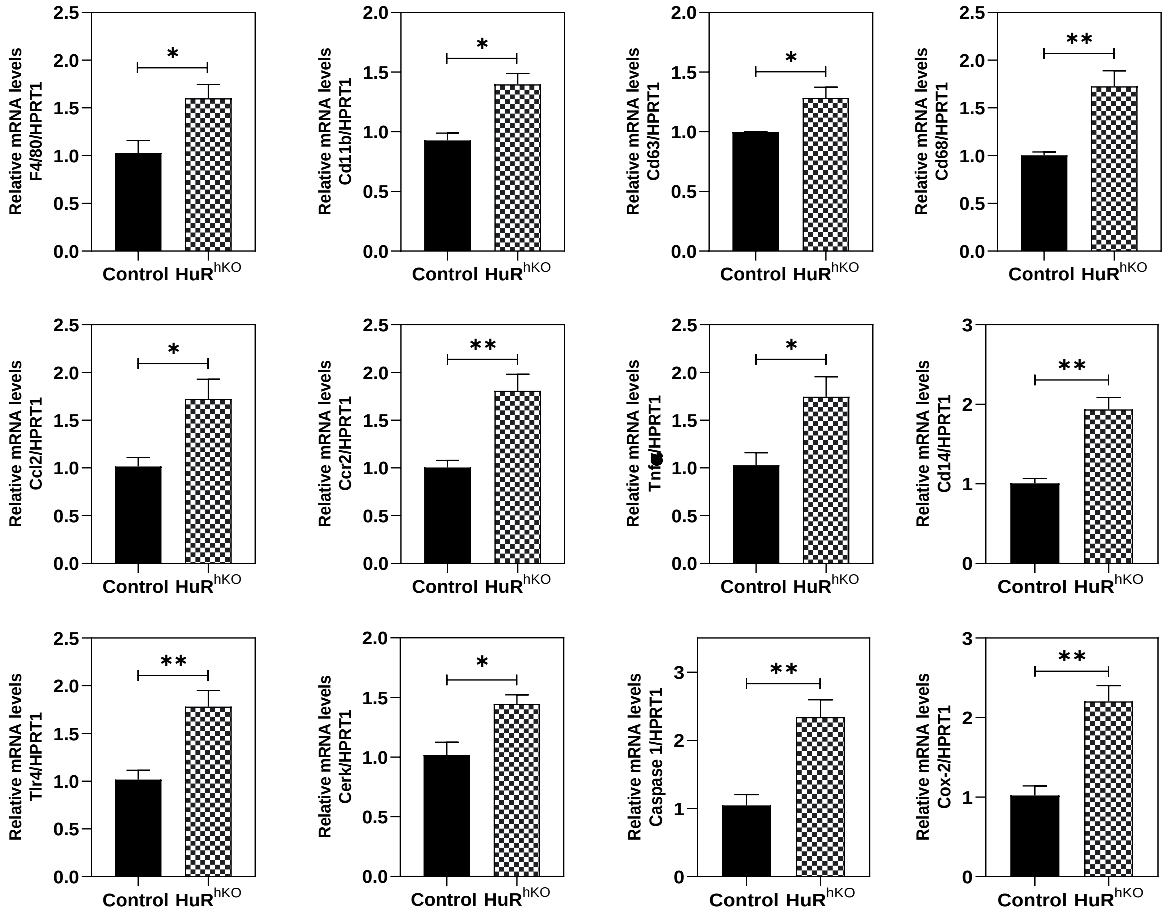

b

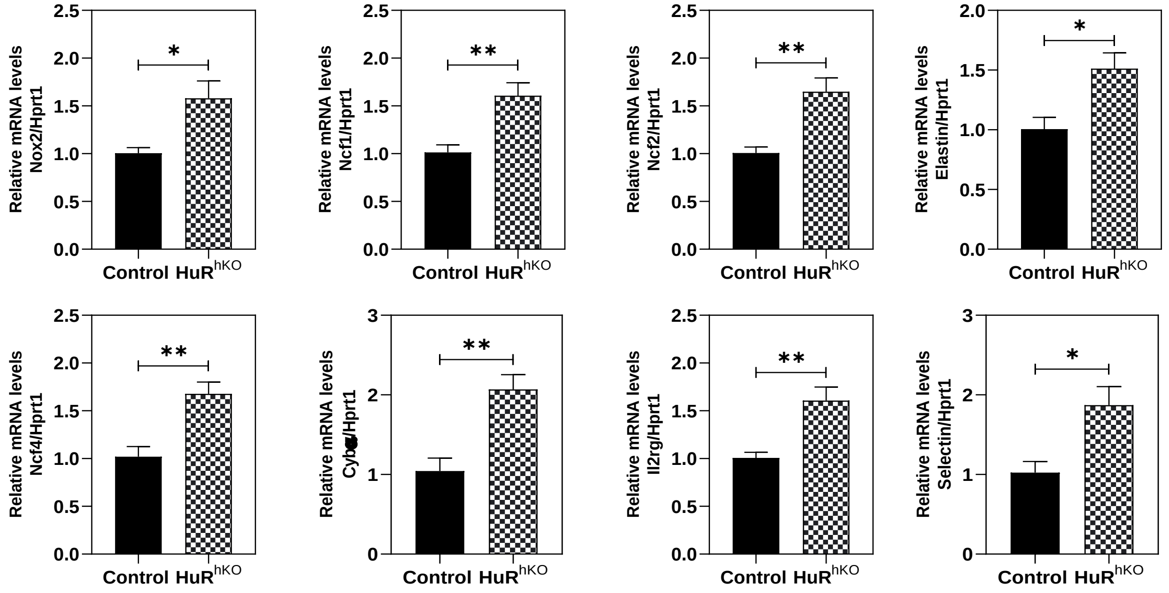

Figure S12

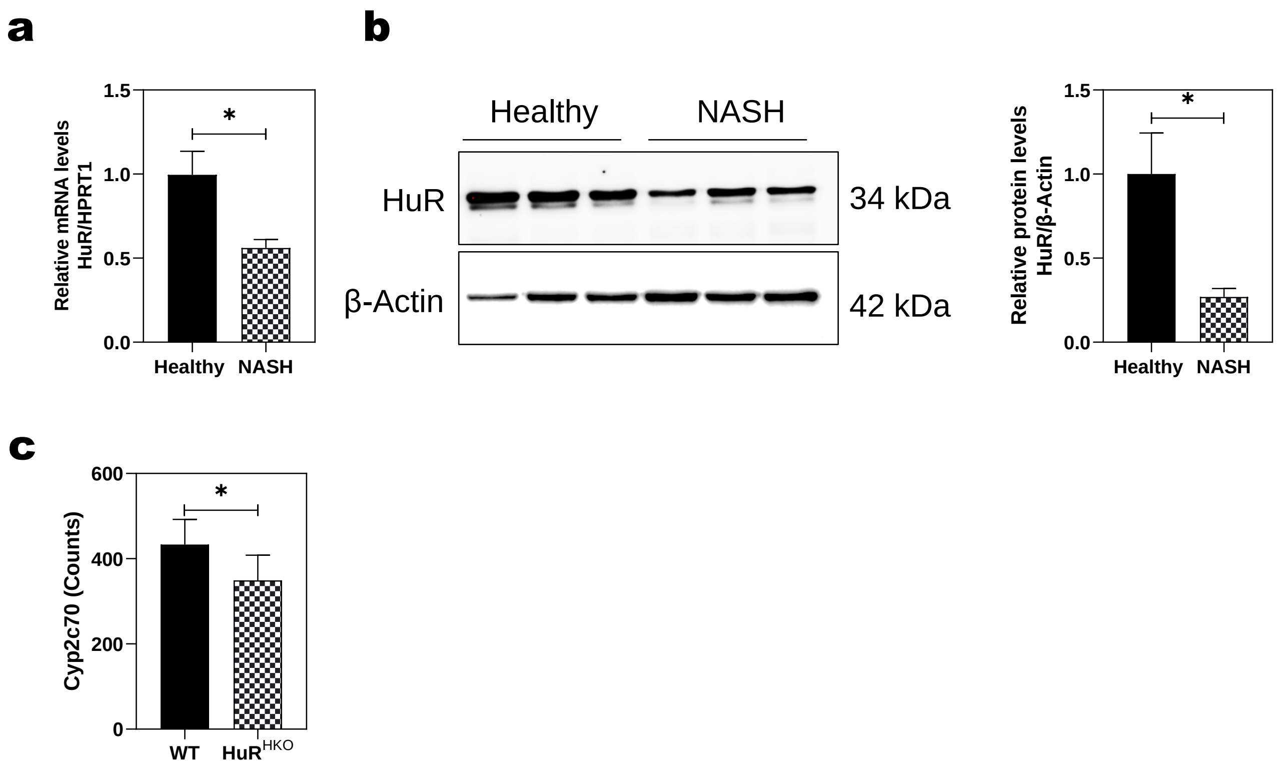
a
Relative mRNA levels
HuR/HPRT1
1.5
1.0
0.5
0.0
*
Healthy
NASH
b
Healthy
NASH
HuR
β-Actin
34 kDa
42 kDa
Relative protein levels
HuR/β-Actin
1.5
1.0
0.5
0.0
*
Healthy
NASH
c
Cyp2c70 (Counts)
600
400
200
0
*
WT
HuR HKO

Raw images

Fig. 5a

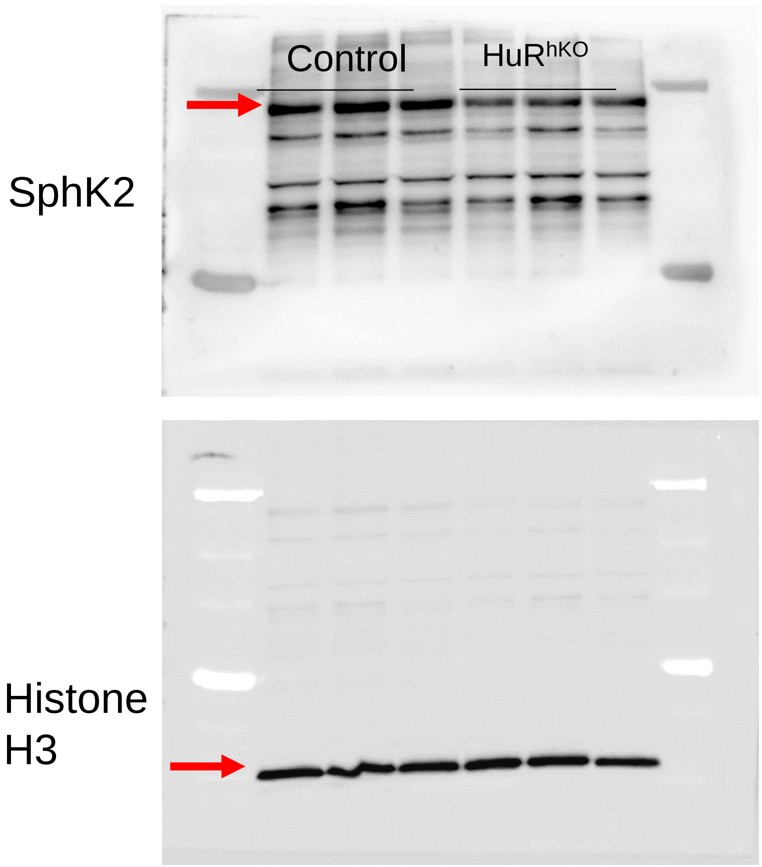

Fig. 5b

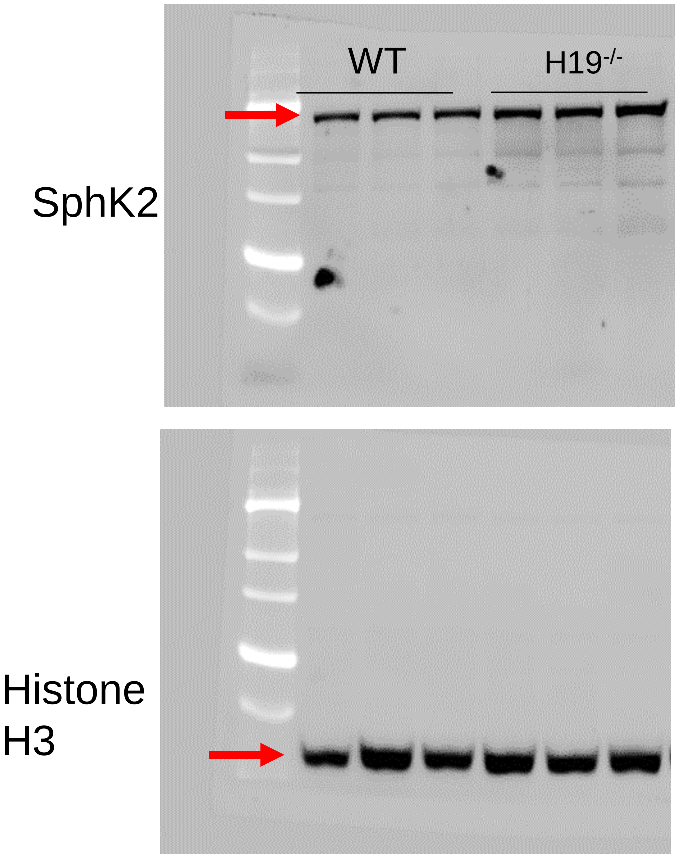

Fig. 5d

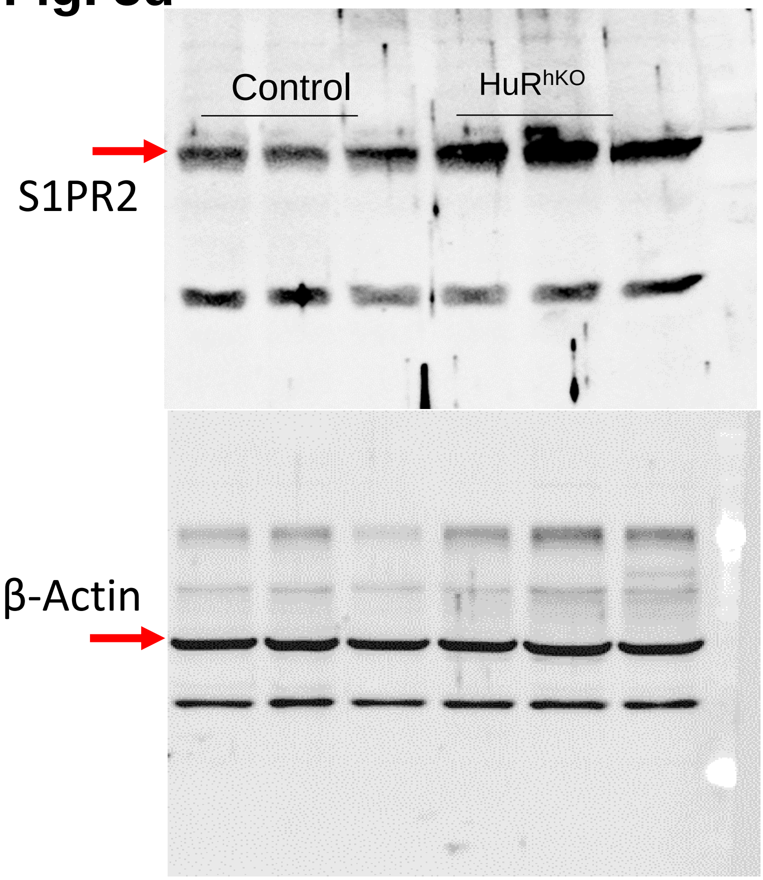

Fig. 8b

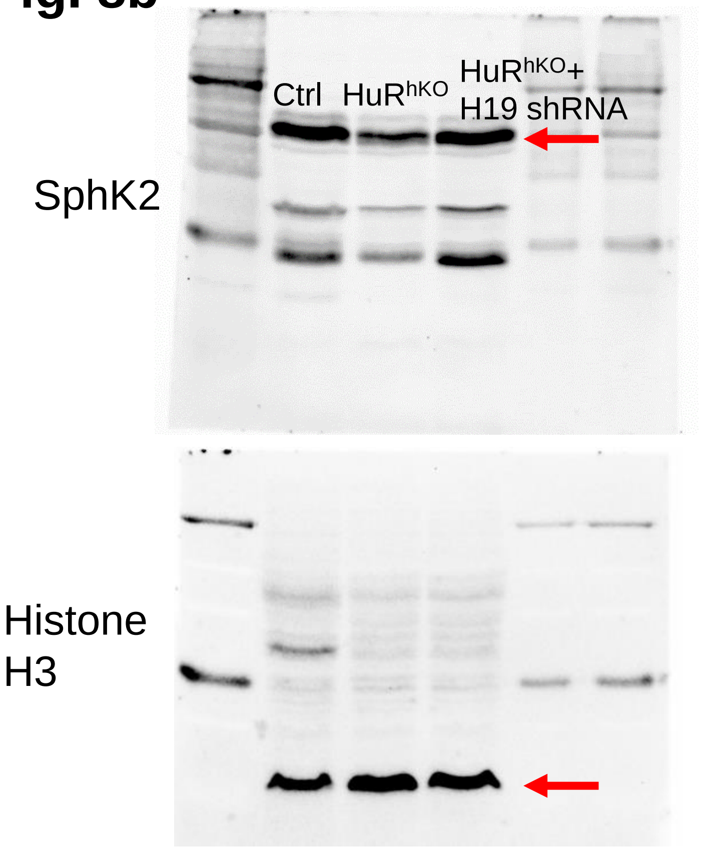

Raw images

Fig. S1b

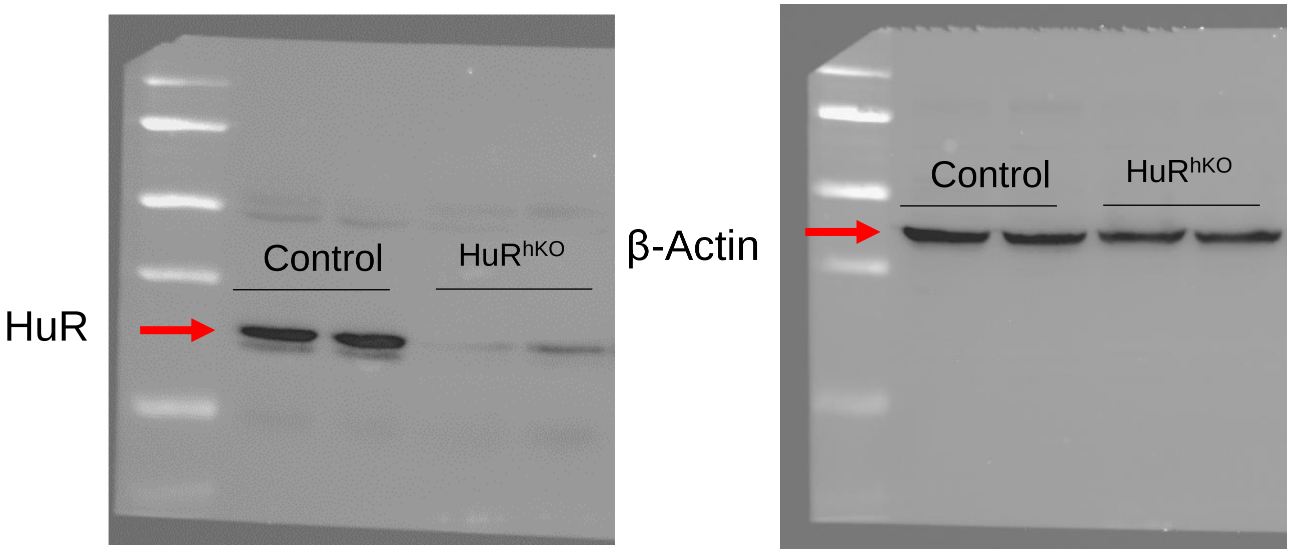

Fig. S6a

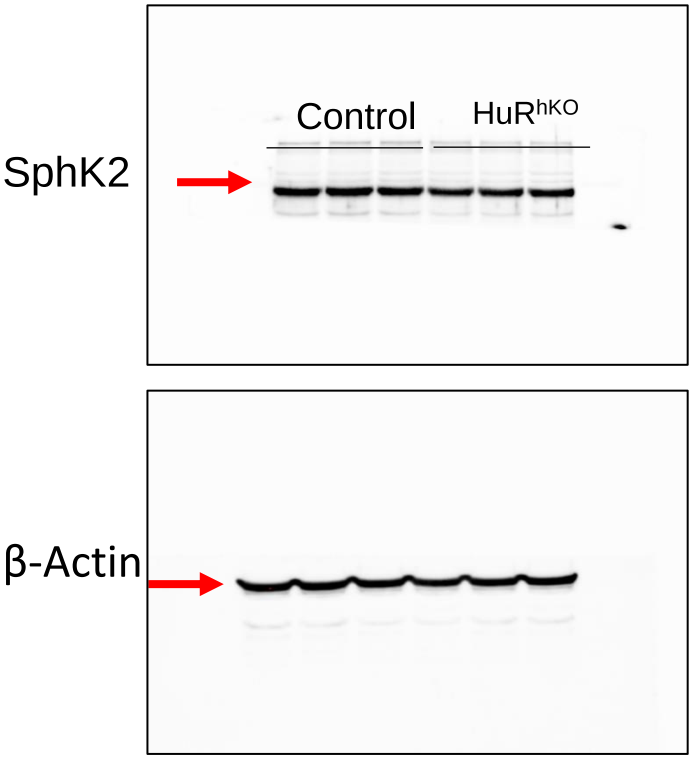

Fig. S6b

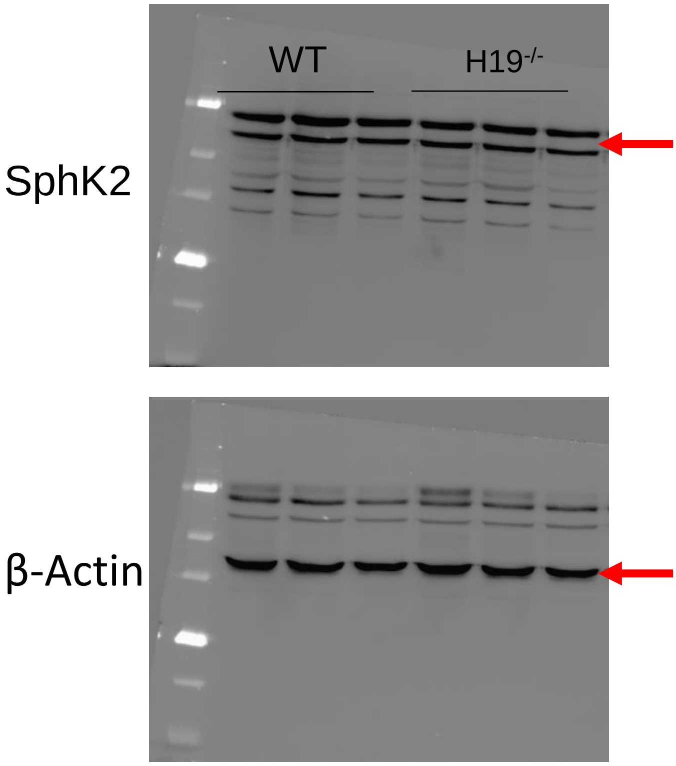

Raw images

Fig. S8a

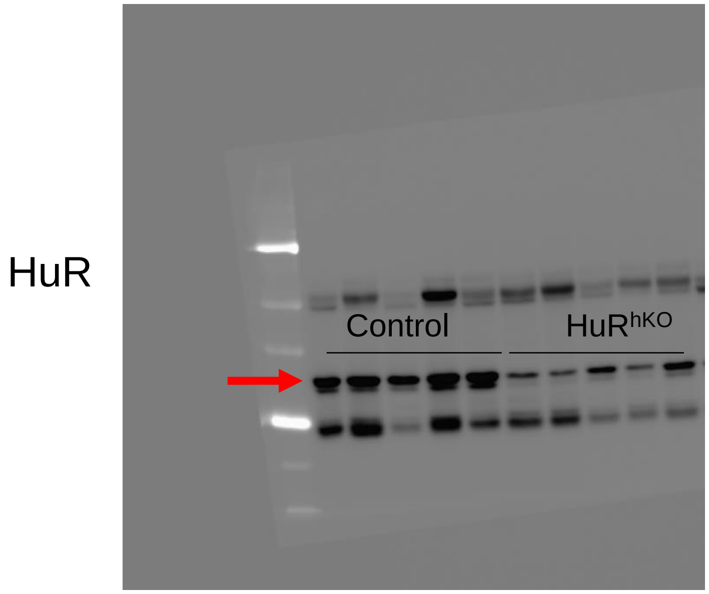

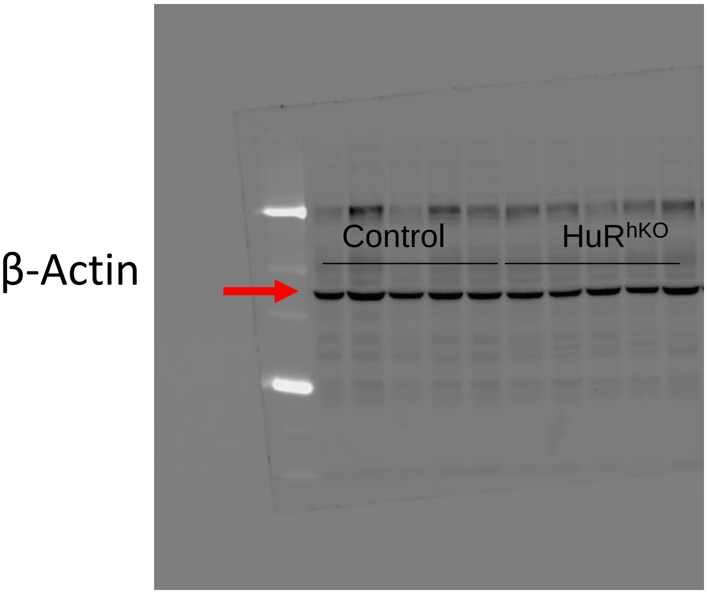

Fig. S10b

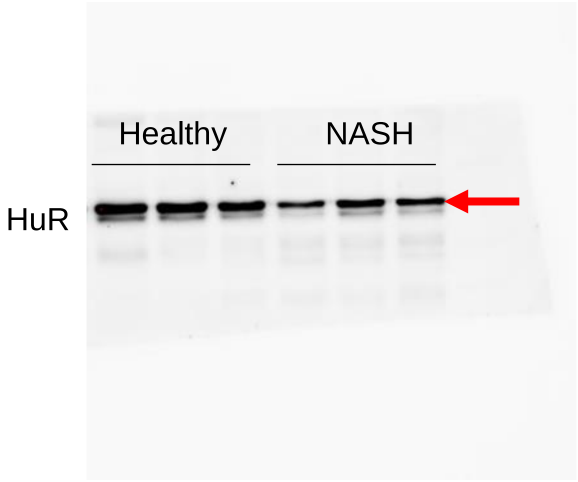

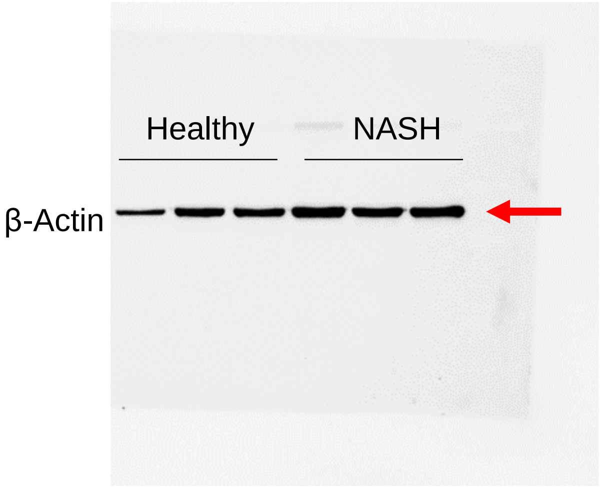

Supplement: Supplementary file 1 — Additional file 1: Additional methods, Supplementary figures, Supplementary tables, and raw image files. The accession number for the raw data FASTQ and processed data file deposit in NCBI is GEO: GSE231215. [file 13578_2022_910_MOESM1_ESM.pdf]
